# THO complex deficiency impairs DNA double-strand break repair via the RNA surveillance kinase SMG-1

**DOI:** 10.1093/nar/gkac472

**Published:** 2022-06-07

**Authors:** Juliette A Kamp, Bennie B L G Lemmens, Ron J Romeijn, Román González-Prieto, Jesper V Olsen, Alfred C O Vertegaal, Robin van Schendel, Marcel Tijsterman

**Affiliations:** Department of Human Genetics, Leiden University Medical Center, Einthovenweg 20, 2333 ZC Leiden, The Netherlands; Department of Human Genetics, Leiden University Medical Center, Einthovenweg 20, 2333 ZC Leiden, The Netherlands; Division of Genome Biology, Department of Medical Biochemistry and Biophysics, Science for Life Laboratory, Karolinska Institutet, SE-17177 Stockholm, Sweden; Department of Human Genetics, Leiden University Medical Center, Einthovenweg 20, 2333 ZC Leiden, The Netherlands; Department of Cell & Chemical Biology, Leiden University Medical Center, Einthovenweg 20, 2333 ZC, Leiden, The Netherlands; Novo Nordisk Foundation Center for Protein Research, University of Copenhagen, Blegdamsvej 3B, DK-2200 Copenhagen, Denmark; Department of Cell & Chemical Biology, Leiden University Medical Center, Einthovenweg 20, 2333 ZC, Leiden, The Netherlands; Department of Human Genetics, Leiden University Medical Center, Einthovenweg 20, 2333 ZC Leiden, The Netherlands; Department of Human Genetics, Leiden University Medical Center, Einthovenweg 20, 2333 ZC Leiden, The Netherlands; Institute of Biology Leiden, Leiden University, Sylviusweg 72, 2333 BE, Leiden, The Netherlands

## Abstract

The integrity and proper expression of genomes are safeguarded by DNA and RNA surveillance pathways. While many RNA surveillance factors have additional functions in the nucleus, little is known about the incidence and physiological impact of converging RNA and DNA signals. Here, using genetic screens and genome-wide analyses, we identified unforeseen SMG-1-dependent crosstalk between RNA surveillance and DNA repair in living animals. Defects in RNA processing, due to viable THO complex or PNN-1 mutations, induce a shift in DNA repair in dividing and non-dividing tissues. Loss of SMG-1, an ATM/ATR-like kinase central to RNA surveillance by nonsense-mediated decay (NMD), restores DNA repair and radio-resistance in THO-deficient animals. Mechanistically, we find SMG-1 and its downstream target SMG-2/UPF1, but not NMD per se, to suppress DNA repair by non-homologous end-joining in favour of single strand annealing. We postulate that moonlighting proteins create short-circuits in vivo, allowing aberrant RNA to redirect DNA repair.

## Graphical Abstract

**Graphical Abstract:**
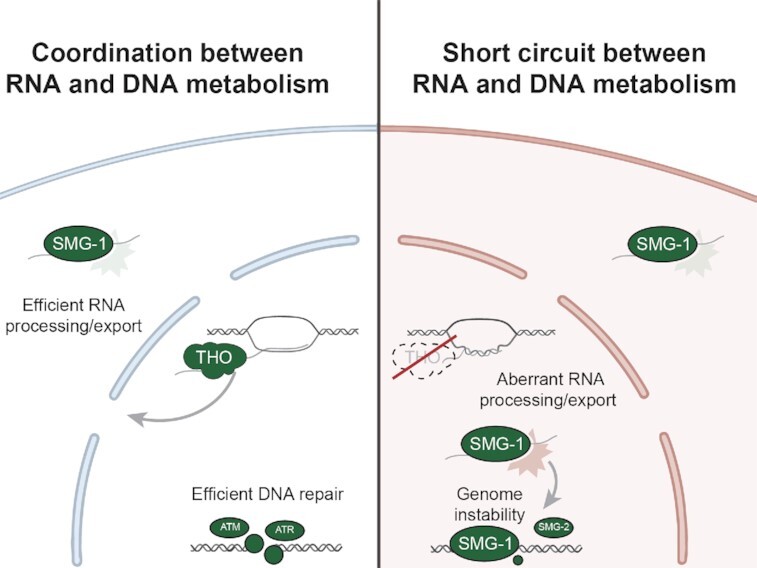
The THO complex and SMG-1 kinase control RNA processing and DNA stability. Viable THO defects cause aberrant SMG-1 activities that compromise DNA double-strand break repair in somatic tissues.

## INTRODUCTION

DNA and RNA molecules are highly effective carriers of genetic information as demonstrated by their ubiquitous use in all domains of life. However, damages to the genetic code are inevitable and even the smallest error can derail cellular fate and impair an individual's fitness, as illustrated by the severe consequences of inherited diseases and cancer predispositions. To safeguard genetic integrity numerous conceptually different DNA and RNA surveillance mechanisms have evolved that detect, remove or resolve aberrant nucleic acid structures. One of the most detrimental damages a genome can encounter is a DNA double-strand break (DSB). In addition to disrupting the sequence of connected nucleic acid bases, DSBs expose fragile moieties within the nucleic acid structure that are vulnerable to nuclease attacks, often causing further loss of genetic information. Repair of DSBs requires multiple complex reactions, which makes it inherently error-prone and/or sensitive to external cues. The mutagenic nature of some DSB repair pathways is a fundamental hazard to human health, causing genetic scars ranging from a single base pair mutation to extensive chromosomal rearrangements involving thousands of base pairs (1). The efficacy of DSBs to cause genetic damage can however also be exploited to improve health. Many anticancer therapies are based on induction of DSBs and DSBs are key intermediates for genome modification and gene therapy approaches (including those based on CRISPR/Cas9 technology).

One of the earliest responses to a DSB is the activation of ATM and/or ATR, two well-conserved members of a family of phosphatidylinositol 3-kinase-related protein kinases (PIKK) that amplify the stress signal, delay cell proliferation and coordinate DNA repair. Recent evidence shows that crosstalk occurs between PIKK family members, and that deregulation of PIKK signalling changes DSB repair pathway choice (2–5). Multiple DNA repair routes have evolved that sense and resolve DSBs depending on the nature of the lesion, the genomic and chromatin environment, the cell cycle stage, and the developmental context. For metazoan cells multiple distinct DSB repair routes have been described: Non-Homologous End-Joining (NHEJ), Homologous Recombination (HR), Single Strand Annealing (SSA), Microhomology Mediated End-Joining (MMEJ) and polymerase Theta-Mediated End-Joining (TMEJ) (1,6). While a versatile toolbox promotes DNA repair flexibility, it also requires strict coordination between the different alternatives. In fact, defective DNA repair pathway choice can be highly toxic and cause lethal levels of chromosomal abnormalities (7). How the different PIKK family members control the relative activities of these DSB repair routes is still poorly understood.

While DNA surveillance mechanisms detect a wide range of DNA damages, they do not detect mistakes in the DNA code (e.g. base changes that do not prevent sealing of the break or distort the DNA helix). However, eukaryotes also evolved RNA surveillance mechanisms that inspect messenger RNA (mRNA) for errors before they have the opportunity to produce faulty proteins in bulk. For instance, the RNA surveillance mechanism called Nonsense-Mediated mRNA Decay (NMD) cleaves and eliminates mRNAs that have a premature termination codon (PTC). PTCs cause mRNAs to encode for truncated proteins that are often non-functional or have dominant-negative effects, making PTCs particularly harmful mutations that cause disease with high penetrance (8). Interestingly, NMD requires a PIKK very similar to ATM/ATR called SMG-1 (Supplementary Figure S1). To trigger the degradation of non-sense mRNAs, SMG-1 phosphorylates the DNA/RNA helicase SMG-2/UPF1, which recruits a protein complex that cleaves the target mRNA. Like ATM and ATR, SMG-1 preferentially phosphorylates serine and threonine residues upstream of glutamine residues (*i.e*. S/TQ motifs). S/TQ motifs are enriched in the NMD target SMG-2/UPF1 but also in many factors involved in genome surveillance and DNA repair. Intriguingly, human ATM and SMG1 phosphorylate identical residues in tumour suppressor p53 and SMG-2/UPF1, and combined loss of ATM and SMG-1 increases haematopoietic cancer incidence (9). This redundancy in phosphorylation targets implies possible signalling interference between NMD and the DNA damage response (DDR). Large-scale proteomic analyses of proteins phosphorylated in response to DNA damage identified over 700 human proteins modified at S/TQ sites, of which surprisingly nearly a third was implicated in RNA processing and transcription (including various RNA splicing and NMD factors) (10). While these phosphorylations are mostly accredited to ATM and ATR activity, the impact of SMG-1 activity might be undervalued and explain the strong bias towards RNA metabolism, given that the list of DNA damage-induced phospho-proteins includes established SMG-1 targets (including SMG-2/UPF1); SMG-1 has similar substrate specificities and can be activated by DNA damage (11). Moreover, SMG-1 deficiency prevents NMD and increases susceptibility to DNA damage in animals (12) as well as plants (13), advocating for a preserved function in both RNA and DNA surveillance.

During the past decades it has become apparent that many enzymes and signalling factors do not perform a single tailored reaction in the cell, but perform additional functions, a phenomenon termed moonlighting. Moonlighting functions can be surprisingly different and often involve different compartments within the cell or organism, which also explains why they are typically discovered by chance or via unbiased screens. While moonlighting proteins provide economical means to expand the range of cellular functions with a limited set of genes, cells have to tolerate the added dimension to cellular complexity and/or evolve regulatory mechanisms to switch between the distinct protein functions. Having moonlighting proteins controlling both RNA and DNA surveillance could promote functional crosstalk, but might also cause signalling conflicts that jeopardize genome stability. To date, surprisingly little is known about the occurrence or molecular impact of such short-circuits *in vivo*.

The existence of shared signalling nodes (like SMG-1) predicts that the cellular abundance, location and/or activity of NMD factors might influence critical processes beyond RNA surveillance. Here, we provide empirical evidence of this prediction, and reveal that mild changes in RNA processing can activate the moonlighting kinase SMG-1, which causes a shift in DNA repair and compromises genome stability. A combination of unbiased DNA repair screens and genome-wide analyses in developing animals allowed us to identify a systemic, potentially harmful link between RNA processing and NHEJ, which is mediated by the PIKK SMG-1.

## MATERIALS AND METHODS

### Genetics

All strains were cultured according to standard C. elegans procedures (14). Alleles used in this study include: LGI: *smg-1(lf238), smg-2(qd101), smg-5(r860)* LGIII: *cku-70(tm1524), cku-80(ok861), lig-4(ok716), brc-1(tm1145), cku-70(lf151), cku-80(lf152), cku-80(lf153), thoc-2(lf158), thoc-5/Y32H12A.2(lf161)*, LGIV:*thoc-7*/B0513.2*(lf160)*, LGV:*pnn-1*/R186.7*(lf159)*,*pnn-1*/R186.7*(ok1872)*, LGX: *pkIs2379* [P*hsp-16.41*::I-SceI-ORF; *rol-6(su1006)*], *pkIS2170* [SSA reporter *Phsp-16.41::ATG::LacZ::ISceI-site::stops::LacZ-ORF*);*unc-119*(+)], *lfIs104* [NHEJ reporter *Pmyo-2::ATG::I-SceI-site: GFP-ORF::LacZ-ORF, Phsp-16.41::mCherry::I-SceI-ORF; rol-6(su1006)*],*lfEx164* [*thoc-2* fosmid WRM0614bD12; *Pmyo-3::mCherry; Prab-3::mCherry*],*lfEx166*[*thoc-7* fosmid WRM0640bD11;*Pmyo-3::mCherry; Prab-3::mCherry*], *lfEx164* [*thoc-5* fosmid WRM0617bE04; *Pmyo-3::mCherry*; *Prab-3::mCherry]*, *lfEx190* [*lig-4* fosmid WRM0634bF07;*Pmyo-3::mCherry; Prab-3::mCherry*], *lfEx195* [*Prpl-28::lig-4*-cDNA;*Pmyo-3::mCherry; Prab-3::mCherry*], *lfEx196* [Prpl-28::lig-4-ORF;*Pmyo-3::mCherry; Prab-3::mCherry*]. All transgenic strains were obtained by microinjection of plasmid/fosmid DNA into the germ line and data presented are from a single representative transgenic line unless noted otherwise. The parental NHEJ reporter transgene IfIs104 was obtained via IR-mediated genomic integration and combined with*pkIs2379* and*pkIs2170* to create the dual reporter strain XF540, which served as the starting strain for the forward genetics screen.

### DSB repair reporter assays

Synchronized L1 animals were obtained by harvesting eggs from hypochlorite-treated gravid adults and overnight starvation in M9 solution (15). Hatched L1 larvae were transferred on NGM plates seeded with either *E. coli* OP50 or HT115 bacteria (16). In order to insure complete RNAi before DSB induction, L1 worms were cultured at 20°C for at least 20 h. Heat-shock driven I-SceI expression was induced by putting the worms at 34°C for 60–180 min, as indicated. After the heat-shock procedure, worms were cultured at 20°C to allow DSB formation, DSB repair and worm development. NHEJ activity was measured by scoring pharyngeal GFP expression using a Leica M165FC fluorescence dissecting-microscope. Experiments were performed in triplicate with 50–200 animals tested for each condition. After GFP quantification, ∼25 adult animals were transferring onto microscope slides with 3% agarose pads and representative pictures were acquired using a Leica DM6000 microscope with 10× objective. SSA activity was measured by scoring animals showing LacZ positive cells in non-pharyngeal somatic tissues (17). One hour prior fixation/LacZ staining, young adults were heat-shocked at 34°C for 120 min to induce SSA reporter expression.

### Forward genetics screen

Dual reporter larvae (XF540) were mutagenized with ethyl methanesulfonate (EMS) using standard procedures (14). Complex F2 populations, each derived from 50 mutagenized P0s, were bleached and synchronized L1 larvae (F3) were seeded on NGM/OP50 plates. On two consecutive days larvae were heat-shocked at 34°C for 180 min in order to maximize GFP ORF correction. GFPlow F3 animals were selected using a Leica M165FC fluorescence dissecting-microscope and clonal F4 populations were tested again for NHEJ activity. Populations showing reduced GFP expression were fixed and stained with X-gal as described previously (17).

### Suppressor screen

Dual reporter larvae defective for *thoc-5* (XF677) were mutagenized with ethyl methanesulfonate (EMS) using standard procedures (14). Complex F2 populations, each derived from 10 mutagenized P0s, were bleached and synchronized L1 larvae (F3) were seeded on NGM/OP50 plates. Larvae were heat-shocked for 140 min, which was determined to be the optimal heat exposure to distinguish all NHEJ proficient worms spiked into a NHEJ deficient population. GFPhigh F3 animals were selected using a Leica M165FC fluorescence dissecting-microscope and clonal F4 populations were tested again for NHEJ activity. Populations showing GFP expression were fixed and stained with X-gal as described previously.

### Positional cloning, genome-wide sequencing and transgenesis

Causal mutations in *thoc-2*, *thoc-5*, *thoc-7* and *smg-1* were mapped by crossing the respective mutants (Bristol) to the related Hawaiian strain CB4856 and performing single-nucleotidepolymorphism mapping on NHEJ proficient versus NHEJ deficient F2 lines (18). Unique EMS-induced genetic alterations in the mapped regions were identified by comparing genome-wide paired-end sequencing data of the parental mutant strains using the Illumina Hiseq 2000 platform, the *C. elegans* reference genome (Wormbase version 225) and MaqGene software (19). Causality was established by complementation analysis using wild-type fosmid arrays. Complemented regions spanned by the fosmids contained only one non-synonymous SNP (Supplementary Figure S17). To create transgenic animals carrying fosmid arrays an injection mix containing 100 ng/μl pBluescript, 10 ng/μl pGH8 (*Prab-3::mCherry::unc-54–3′UTR), 5 ng/μl pCFJ104 (Pmyo-3::mCherry::unc-54–3′UTR)* and 10–50 ng/μl fosmid DNA *(lig-4 WRM0634bF07, thoc-2 WRM0614bD12, thoc-7 WRM0640bD11, thoc-5 WRM0617bE04, pnn-1 WRM0637aA06*) was injected into the gonads of young adults. For *lig-4* cDNA and *lig-4* ORF containing vectors, 5 ng/μl plasmid DNA was added instead of the fosmid DNA.

### IR sensitivity assays

All IR experiments were performed with a dose rate of 10–15 Gy/min using an electronic X-ray generator (XYLON International). Figures provide mean values of three independent experiments. L4 assay: three L4 animals per plate were treated with various doses of IR (three plates per condition) and were removed after an egg-laying period of two days. Progeny survival was scored 1 day after removal of the irradiated p0. L1 assay: ∼200 L1 larvae per plate were treated with various doses of IR (three plates per condition) and vulva phenotypes were scored 5 days post IR. Representative pictures of irradiated populations were acquired using a Leica DFC295 camera/M165FC microscope.

### Transcriptome sequencing

To obtain clean L1 populations and remove dead corpses, o/n starved L1 progeny from hypochlorite-treated gravid adults were filtered using 10 μm nylon filters (Millipore). Total RNA of >3000 L1s per sample was extracted as follows: L1 animas were collected in 100 μl M9, 400 μl Trizol was added, vortexed 2 min, followed by 4 snap freeze/thaw cycles at –196°C/37°C, 200 μl Trizol was added, incubated 5 min RT, 120μl chloroform was added, incubated 2 min at RT, centrifuged 15 min at 4°C (16 000 rcf), 350 μl supernatant was mixed with 70% ethanol (1:1) and total RNA was purified using Purelink^®^ RNA columns (Ambion) and stored at −80°C. Total RNA was DNAse treated (Turbo DNA-free, Ambion) and RNA quality was verified using RNA 6000 Pico kit (Agilent). RNA-Seq was performed on an Illumina HiSeq 2000 platform using standard reagents. Raw reads were aligned by TopHat on Wormbase assembly 238, which allows for reads to be split over splice junctions. Next, we used DESeq to identify differentially expressed transcripts (*q* ≤ 0.05) (20). For differential exon expression, DEXSeq was used using standard settings (*P*_adjust_ ≤ 0.05) (21). For each mutant two or three samples were sequenced to account for variation between isogenic L1 populations. rMATS was used to determine alternatively spliced events. Since rMATS is unable to intron retention in introns that are not part of annotated alternative transcripts we also analyzed the data using iReads (22,23), which is specifically designed to find retained introns in introns that do not overlap annotated exons.

### PCR-based DSB repair assay

Synchronized L1 larvae were seeded on NGM/OP50 plates and heat-shocked at 34°C for 180 min. Heat-shock was repeated 24 h after seeding. Expression of mCherry-tagged ISceI was verified using a Leica M165FC fluorescence dissecting-microscope. Genomic DNA was extracted 24 h after the second heat shock using a DNeasy Blood & Tissue kit (Qiagen). The first PCR on 18.5 ng isolated DNA was performed using Phusion High Fidelity Polymerase (ThermoFisher). Primers (forward: 5′ AAAGTTATCTCCAGGCTCGC, reverse: 5′ TTCACCCTCTCCACTGACAG) containing 5′ adaptor sequences (forward: 5′ GATGTGTATAAGAGACAG, reverse: 5′ CGTGTGCTCTTCCGATCT) flanking the I-SceI target site of the NHEJ reporter were used. PCR products were purified using Ampure XP beads(Beckman Coulter). A second PCR of 5 cycles was performed to barcode the products using p5 and p7 index primers. Final PCR products were purified using Ampure XP beads (Beckman Coulter) and sequenced on an Illumina NovaSeq 6000. Reads were filtered and aligned to a reference sequence using a custom JAVA program (available upon request). Only reads of which both the start and end of the sequence lied within the specified primers were used. The custom JAVA program was used to assign the reads to different classifications: wildtype, SNVs, deletion, insertion, delins (deletion with insertion), tandem duplication (TD) or compound TD (TD with an additional mutation). Reads containing variation in the number of adenines in a polyA-tract were filtered and reads containing consequences of mutagenic repair (deletions, insertions, delins, TDs and compound TDs) were plotted using R.

### Phosphoproteome analysis

Worm populations from twenty plates per genotype were rinsed off and washed with M9 solution (15). Lysis buffer (6M GdmCl (Guanidine hydrochloride), 100 mM Tris pH 8.5, 10 mM TCEP (Tris (2-carboxyethyl)phosphine), 40mM CAA (2-Chloroacetamide) and 4 μl benzonase (250 U/μl, Novagen) was added after the populations were washed with autoclaved demi water. Worms were snap-frozen in liquid nitrogen and stored at −80°C. After thawing, worms were sonicated four times for 30 s in an ice-filled water bath. Lysis of worms was verified using a dissection microscope. Lysates were snap-frozen in liquid nitrogen. Lysates were thawed and protein concentrations were measured using a BCA kit (Thermo Scientific). Proteins were digested using Trypsin/Lys-C mix (Promega) and phosphopeptides were enriched, cleaned and eluted as described previously (24). Samples were analyzed using a Q-Exactive HF mass spectrometer. Phosphosites identified in all samples were included for statistical analysis and compared between genotypes using two-sided *t*-tests. Significance of global phosphorylation changes was determined by permutation of the set.

### RNA:DNA hybrid staining

Genomic DNA was isolated from worm populations grown on a NGM plate using a Blood and Tissue Culture Kit (Qiagen), without the RNAse A treatment step. Concentrations of DNA were measured using Qubit. Equal amounts of DNA per sample were treated with RNAse H (NEB) or mock-treated at 37°C for 1 h. Samples were diluted in TE buffer with 6× SSC and blotted on a nitrocellulose membrane. The membrane is washed with TE0.1 and baked at 65°C for 1 h. The membrane is blocked in 5% milk in PBST overnight. Staining is performed with 1:3000 s9.6 antibody (Kerafast) in 3% milk 1% BSA in PBST for 2 h at room temperature. After washing (once 3% milk 1% BSA in PBST, twice with 1% BSA in PBST), the membrane was stained with anti-mouse^HRP^ for 1 h at room temperature, washed (thrice 1% BSA in PBST, twice with PBST and once with PBS) and visualized using enhanced chemiluminescence. Dot intensities were quantified using ImageJ.

### Fecundity assessment

L4 larvae (p0) were isolated on seeded NGM plates and transferred every 24 h until no eggs were laid (day 5). Larvae and dead eggs on NGM plates were counted 24 h after transfer of the p0 worm. Brood size was calculated as the sum of larvae and eggs on all plates per p0. Survival was calculated by dividing the number of alive worms by the total brood size.

## RESULTS

### Dual reporter system to measure NHEJ activity in *Caenorhabditis elegans*

**Figure 1.**
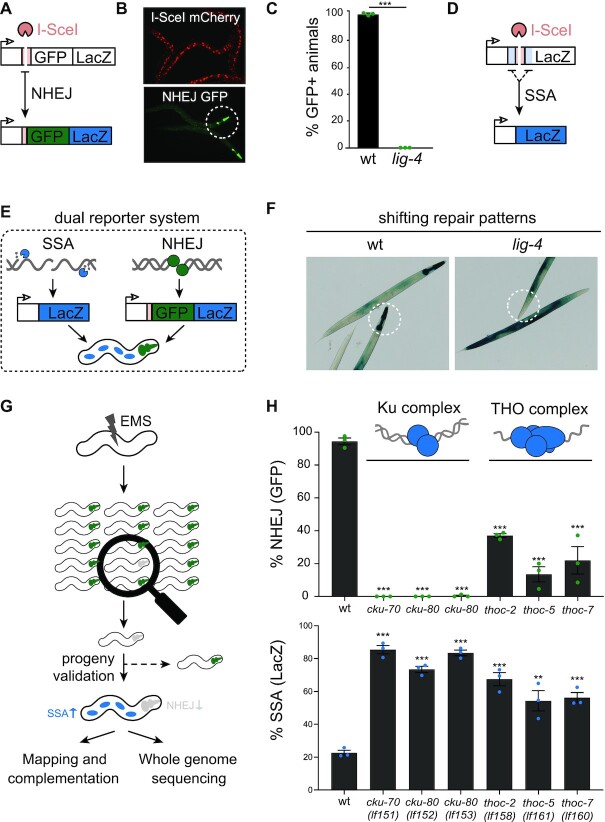
*In vivo* NHEJ reporter identifies role of Ku and THO complex in DSB repair. (**A**) Schematic diagram of our NHEJ reporter based on heat-shock-inducible expression of mCherry::ISceI and break induction at the ISceI target site in a multi-copy integrated reporter transgene. Mutagenic NHEJ is measured by GFP/LacZ ORF correction. (**B**) Representative pictures of animals expressing nuclear mCherry::I-SceI (6 h post heat-shock), pharyngeal GFP (3 days post heat-shock). Circle indicates a representative pharynx. (**C**) Quantification of GFP-positive pharynges in NHEJ-proficient wild-type and NHEJ-deficient *lig-4* deficient reporter animals, heat-shocked for 180 min at L1 stage and measured in adults. Average of three populations (*n* > 200) is depicted. Dots indicate the average of each experiment (two-tailed t-tests *P < 0.05; **P < 0.01, ***P < 0.001). (**D**) Schematic diagram of the Singe Strand Annealing (SSA) reporter that serves as an indirect NHEJ activity assessment tool. Heat-shock-inducible expression of mCherry::ISceI leads to break induction at the I-SceI target site in a multi-copy integrated reporter transgene. SSA is measured by LacZ ORF correction that is established by repair of the induced break using annealing of flanking stretches of homologous nucleotides. (**E**) Schematic diagram of the dual reporter system. The NHEJ reporter is expressed in non-dividing pharyngeal muscle cells, leading to GFP and LacZ production in the pharynx of NHEJ-proficient nematodes after break induction and erroneous repair. The SSA reporter is expressed in dividing somatic cells, leading to LacZ expression in the soma after SSA of induced breaks. (**F**) Representative pictures NHEJ-proficient (wild-type) and NHEJ-deficient (*lig-4*) dual reporter animals. In NHEJ-proficient animals, NHEJ reporter activity is visible as LacZ staining throughout the pharynx, while SSA staining is low in the dividing somatic cells in the body. In NHEJ-deficient animals, LacZ staining is absent in the pharynx, but SSA activity is increased in replicating cells, leading to increased LacZ staining in the soma. Circles indicate representative pharynges. (**G**) Forward genetics screen set-up. Genomes of animals carrying the dual reporter system were mutagenized using ethyl methanesulfonate (EMS). NHEJ activity of the progeny was assessed using the NHEJ reporter and GFP-negative worms were isolated. Reporter animals of which NHEJ impairment was verified using LacZ staining were followed up to identify causal mutations. (**H**) Quantification of GFP-positive pharynges and non-pharyngeal LacZ staining of identified Ku or THO complex mutants and wild-type animals. Average of three populations (*n* > 200) is depicted. Dots indicate the average of each experiment. Error bars represent SEM. Asterisks indicate statistically significant difference compared to wildtype controls (two-tailed *t*-tests *P < 0.05; **P < 0.01, ***P < 0.001).

In recent years, *C. elegans* has become a powerful model to study DNA damage responses in a developmental context, leading to the discovery of new DSB repair mechanisms in somatic as well as germline tissues (25,26). While germline tissues strictly use homology-directed DSB repair (27,28), somatic tissues use different and partly competing repair routes depending on the nature of the break and the proliferative state of the tissue (7,17,29). NHEJ is considered the principle DSB repair route in somatic tissues, yet little is known about its regulation *in vivo* (29). A key obstacle in NHEJ research is the existence of alternative end-joining mechanisms, which mask the defects in NHEJ activity and obscure interpretations of DSB repair assays. To study DSB repair in somatic tissues we previously created animals in which we induced site-specific DSBs in pathway-specific reporter systems (17,30). Here, we set out to create a system able to directly detect NHEJ efficacy in single animals and perform an unbiased search for NHEJ regulators. We designed a fluorescent reporter system based on two key features of classical NHEJ: its error-prone nature (often leaving small deletions/insertions) and its prevalence in non-dividing tissues. In brief, the NHEJ reporter is based on mutagenic repair of a targeted DSB and consequent restored expression of a downstream GFP/LacZ open reading frame (Figure 1A). NHEJ reporter expression is driven by a tissue-specific *myo-2* promoter, limiting GFP/LacZ expression to pharyngeal muscle cells. Pharyngeal muscle cells are terminally differentiated at the first larval stage and thus rely heavily on classical NHEJ (29). Site-directed DSB induction was achieved using a heat-shock inducible, mCherry-tagged I-SceI endonuclease (Figure 1B). One day after I-SceI induction, nearly 100% of the animals carrying a multicopy NHEJ reporter transgene showed GFP expression, which was fully dependent on the classical NHEJ factor LIG-4 (Figure 1C). The robust induction of GFP expression and the large difference in NHEJ activity between the genetic conditions provides a solid window for an unbiased search for NHEJ modulators. We reasoned, however, that whilst loss of GFP expression could reflect a specific loss of NHEJ, it could also reflect reduced I-SceI expression, loss of the reporter, or other general defects in DSB induction or repair. To discriminate between these scenarios and find bona fide NHEJ regulators, we made use of a previous observation demonstrating that defects in somatic NHEJ result in a stark increase in compensating DSB repair pathways such as SSA, provided that the DSBs is surrounded by stretches of identical sequence (17). We thus generated a more advanced NHEJ reporter animal that carries both the reporter specific to monitoring NHEJ as well as a well-characterized SSA reporter (17) (Figure 1D). These dual reporter animals detect NHEJ activity directly, by measuring NHEJ in nonreplicating pharyngeal cells, as well as indirectly, by measuring SSA in various replicating somatic tissues (Figure 1E). As predicted, loss of NHEJ activity in these animals resulted in a dramatic shift in LacZ staining pattern (Figure 1F). While control animals showed robust LacZ staining in the pharynx but rarely in other somatic tissues (reflecting relatively high NHEJ and low SSA activities, respectively), *lig-4* mutants showed no LacZ staining in the pharynx but strong LacZ restoration in other somatic tissues (reflecting NHEJ deficiency and an ensuing shift towards SSA). As both the NHEJ and SSA readouts depend on I-SceI activity, shifts in LacZ patterns are indicative of changes post DSB induction and also discriminate general DSB repair defects from specific changes in NHEJ activity.

### Identification of novel regulators of NHEJ

Having validated the reagents, we next searched for genetic conditions that control NHEJ activity. To this end, we performed a forward genetics screen in which we induced random genomic mutations in dual reporter animals by ethyl methanesulfonate (EMS) and assessed NHEJ activity among mutant progeny (i.e. complex F3 populations were screened for animals with reduced pharyngeal GFP signal) (Figure 1G). These ‘GFP-low’ animals were selected and their clonal progeny was again tested for NHEJ activity to identify heritable traits that affect pharyngeal GFP expression. To exclude false-positive NHEJ mutants we checked for unaffected mCherry-I-SceI expression and monitored SSA activity in all mutant candidates. Only NHEJ mutants that also showed increased SSA activity (indicative of a specific repair defects post DSB induction) were selected for further analysis, including genetic complementation assays and whole-genome sequencing (Figure 1G). By screening ∼9000 unique haploid genomes we found seven *bona fide* NHEJ mutants: four showing reduced GFP expression and three showing no pharyngeal GFP at all. We quantified both NHEJ activity (GFP expression) and SSA activity (somatic LacZ expression) of synchronized clonal populations and observed the expected inverse correlation between NHEJ defect severity and increased compensatory SSA activity (Figure 1H). By combining whole-genome sequencing and classical complementation assays using null alleles of the known NHEJ genes *lig-4, cku-80* and *cku-70*, we identified the causal mutations in the three mutants devoid of NHEJ activity: alleles *lf152* and *lf153* were caused by nonsense mutations in *cku-80* and allele *lf151* was caused by a nonsense mutation in *cku-70* (Supplementary Figure S2). The identification of the canonical NHEJ complex Ku using this unbiased approach validated both our dual reporter system as well as our screening setup. Interestingly, the modifier alleles *lf158*, *lf159*, *lf160* and *lf161* were not caused by mutations in known canonical NHEJ genes (Supplementary Figure S3). To identify the chromosomal regions linked to the NHEJ defects we performed classical positional mapping using single nucleotide polymorphisms (SNPs) between the Bristol dual reporter mutants and a Hawaiian mapping strain CB4856. In parallel, we performed genome-wide sequencing to find non-synonymous SNPs in the mapped regions. Strikingly, we found that three out of four modifier mutants mapped to a subunit of the very same multi-protein complex, the THO complex, strongly suggesting that defective THO function causes reduced NHEJ activity. Indeed, rescue experiments using extrachromosomal expression arrays confirmed that the phenotypic consequences of alleles *lf158*, *lf160* and *lf161* were caused by defective THOC-2, THOC-7 and THOC-5 function, respectively (Supplementary Figures S4 and S17). THOC-2 depletion by RNA interference also mirrored the changes in DSB repair (Supplementary Figure S4). THO is a well-conserved RNA binding complex that controls processing and nuclear export of mRNA (31). THO also has been implicated in safeguarding genome integrity, yet the mechanisms underlying the latter are still unclear (32). THO deficiency causes genome instability in part through increased levels of RNA:DNA hybrids (or R-loops), which in dividing cells cause impaired DNA replication and DNA damage (32–36). Notably, our data indicate that THO deficiency can change DNA repair in non-dividing cells, and thus may trigger genome instability independent of DNA replication.

### THO deficiency impairs NHEJ and RNA processing

**Figure 2.**
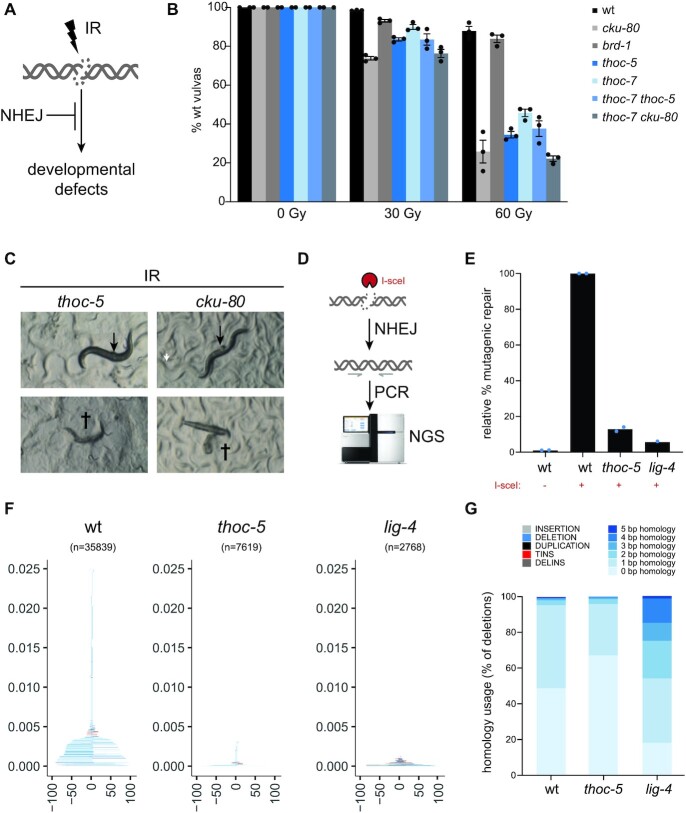
THO deficiency causes radiation sensitivity and DNA end joining defects. (**A**) NHEJ protects nematodes from ionizing irradiation (IR)-induced developmental defects, including abnormal development of the vulva. (**B**) Larvae were challenged with indicated doses of IR and vulva development was scored. Values depict averages of three independent experiments and error bars represent S.E.M. Dots indicate the average of each experiment. (**C**) Examples of defective vulva development as a consequence of IR in *thoc-5* and *cku-80* animals. Arrows show protruding vulvas, crosses indicate lethality due to germline extrusions or internal hatching of progeny. (**D**) Schematic representation of PCR-based assay to quantify mutagenic repair. Breaks were induced at the NHEJ reporter by expressing I-SceI. Genomic DNA was isolated and PCR reactions are carried out with primers surrounding the break site. Amplicons were analyzed using next generation sequencing (NGS). (**E**) Quantification of mutagenic DSB repair. Repair efficiency was normalized to the number of mutagenic repair events after I-SceI induction in wildtype worms. Dots represent averages of each replicate. (**F**) Tornado plots depict type and size of mutagenic repair events identified by NGS (n indicates number of mutagenic events). Color code indicates type of event as in Figure 2G. (**G**) Relative distribution of deletion types found by NGS in wildtype, *thoc-5* or *lig-4* reporter animals.

To verify if the THO complex is needed for efficient repair of DSBs throughout the genome (and independent of I-SceI, GFP or LacZ expression), we exposed the mutant animals to ionizing radiation (IR). IR causes DSBs in a dose-dependent manner and can be used to study tissue-specific DSB repair (37). Different tissues have different DNA repair capacities and thus IR tolerance needs to be examined in a tissue-specific manner. In *C. elegans*, IR tolerance of germ cells can be examined via a so-called ‘L4 assay’, while somatic tissues can be tested via a ‘L1 assay’ (Supplementary Figure S5). In a L4 assay, germ cells of adolescent L4 larva are irradiated and progeny survival is analysed, while in a L1 assay synchronized L1 larva are irradiated and tracked for developmental defects (often focussing on well-defined organs such as the vulva, of which the precursor cells are not replicating during the L1 stage). Animals lacking canonical NHEJ are exceptionally sensitive to IR in the L1 assay, but not in the L4 assay, reflecting the key role of NHEJ in somatic cells (Figure 2A and (29)). In contrast, mutants defective in HR-mediated repair are typically hypersensitive to IR in the L4 assay, consistent with the strong HR bias in germ cells (29,30,38). Indeed, we find animals deficient for BRD-1 (ortholog of the human HR factor BARD1) to be hypersensitive to IR in the L4 assay, while animals lacking the NHEJ factor CKU-80 behaved like wild-type animals (Supplementary Figure S5). Similar to canonical NHEJ mutants, germ cells of *thoc-5* and *thoc-7* animals did not show hypersensitivity to IR, indicating that defective THO function does not sensitize genomes to IR *per se* (Supplementary Figure S5). Yet, when we subjected these animals to the L1 assay, we observed the inverse pattern. While irradiated *brd-1* mutants showed only minor somatic defects, irradiated *cku-80*, *thoc-5* and *thoc-7* mutants showed acute sensitivity, resulting into ∼75%, ∼65% and ∼60% of animals having defective vulva development upon 60 Gy of IR, respectively, while only ∼10% of wild-type animals was affected at this dose (Figure 2B). Analogous to *cku-80* mutants, irradiated *thoc-5* mutants developed various IR-dependent somatic defects, including protruding vulvas, ruptured vulvas and so-called ‘bag-of-worms’ where progeny hatches within the mother because of an egg laying defect (Figure 2C).

Epistasis analysis revealed that the *thoc-5* single mutant and *thoc-5 thoc-7* double mutant are equally sensitive to IR, implying that THOC-5 and THOC-7 act in the same pathway/complex. Similarly, *cku-80* single mutants and *cku-80 thoc-7* double mutants are equally sensitive to IR, indicating that the THO complex affects IR tolerance via NHEJ. Given that *thoc-7* deficiency does not increase the number of somatic defects in NHEJ null mutants (with or without IR), viable THO mutations appear to change DSB repair pathway choice without causing a large number of extra DSBs. In accordance with that notion, we find that the increased frequency of SSA in *thoc-7* mutants relies on I-SceI induced DSBs, and thus is not caused by more spontaneous DSBs (Supplementary Figure S6).

The GFP reporter and IR studies also demonstrate that impaired THO function alters NHEJ efficacy in pharyngeal cells as well as vulval precursor cells (but not in germ cells), which together with the data from the somatic SSA reporter hints towards the existence of an unexplored near-systemic modulator of DNA repair fidelity. Our transgenic NHEJ reporter system creates targeted DSBs in nearly all somatic tissues, but only in pharyngeal cells this will be translated into GFP expression. We reasoned that THO deficiency likely altered DSB repair in many more cells than was visualized by the NHEJ reporter. To test this hypothesis, we developed a systemic PCR-based assay to quantify mutagenic repair at the I-SceI target site. In short, I-SceI expression was induced in synchronized L1 larva and DNA repair products were PCR-amplified and subjected to next-generation sequencing analysis (Figure 2D). In line with a widespread defect in NHEJ, both *lig-4* and *thoc-5* mutants showed a marked drop in the frequency of NHEJ-associated repair footprints (Figure 2E and F). Interestingly, *lig-4* mutant animals show a significant shift towards micro-homology driven DSB repair (i.e. 1–10 bp homology at break junctions) while *thoc-5* mutants do not, suggesting that *thoc-5* deficiency causes a defect in NHEJ as well as micro-homology-driven repair routes such as TMEJ (Figure 2G). Thus DNA repair footprint analyses as well as the IR sensitivity studies indicate that THO deficiency causes a significant but selective defect in DSB repair, which limits NHEJ (and potentially TMEJ) but allow SSA and meiotic recombination – the latter a thoc-5/thoc-7 mutants display normal fertility and intact diakinesis chromosomes (Supplementary Figure S5 and S7).

**Figure 3.**
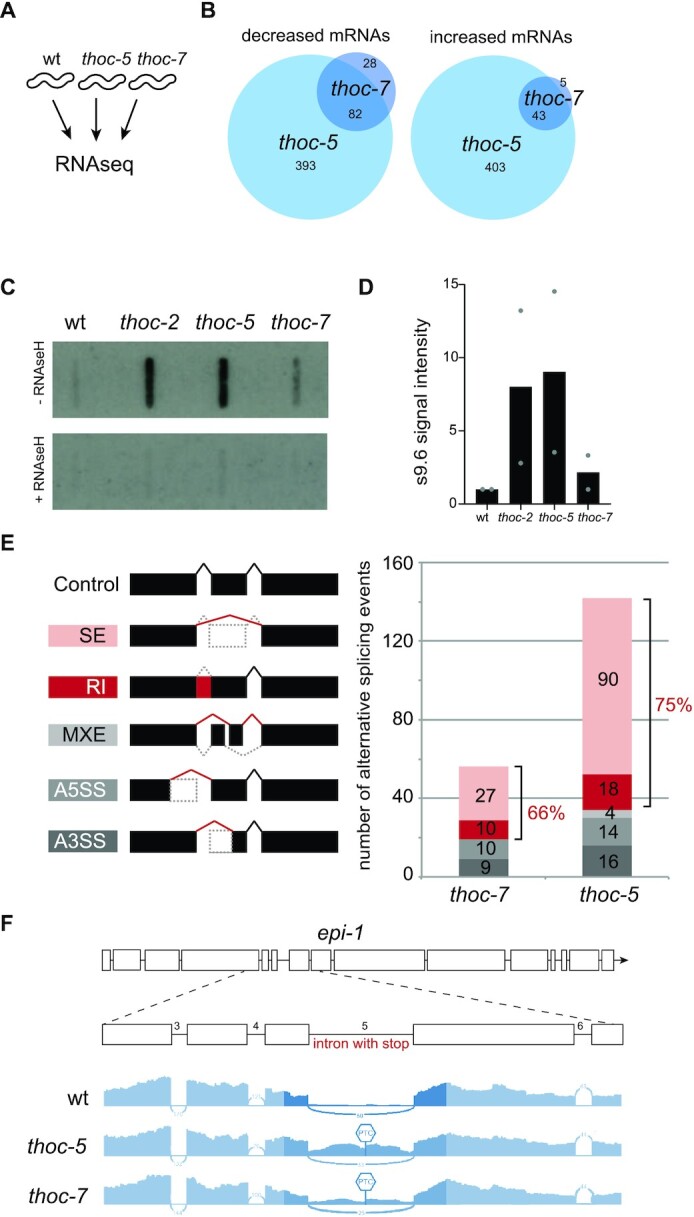
THO deficient animals suffer from RNA splicing errors and R-loops. (**A**) RNA sequencing and direct comparison of NHEJ-deficient THO mutants. (**B**) Venn diagrams of significantly (*q* ≤ 0.05) downregulated transcripts (left) and significantly (*q* ≤ 0.05) upregulated transcripts (right) identified using RNA sequencing. (**C**) Representative slot blots to quantify R-loops in THO mutants. Nucleic acid extracts of THO mutant worms show increased s9.6 antibody staining. Specificity of the s9.6 antibody signal was confirmed by RNAse H treatment to digest DNA:RNA hybrids. (**D**) Quantification of R-loop levels. s9.6 antibody signal intensity was normalized to WT and RNAse-H digested controls. Dots represent values of each experiment. (**E**). Schematic representation of the different splicing events detected by rMATS: SE, skipped exon; RI, retained intron; MXE, mutually exclusive exon; A5SS, alternate 5′ splice site; A3SS, alternate 3′ splice site. Bar chart indicates number of splicing events detected and altered in *thoc-5* or *thoc-7* animals compared to wildtype controls. Color code is the same as in schematic on the left. (**F**) Typical example of a selective splicing error in THO mutants, causing a retained intron and robust expression of PTC-bearing mRNAs. Gene model of *epi-1* and below (in blue) simplified sashimi plots for each genotype. Sashimi plots depict relative RNAseq read-depth around the highlighted region of *epi-1*; both *thoc-5* and *thoc-7* mutants selectively retain the fifth intron of *epi-1* transcripts. The predicted position of the first PTC is indicted.

Given the established role of THO in mRNA processing we searched the transcriptome for clues on how this complex might skew DNA repair activities. We performed RNAseq analysis on synchronized L1 animals and identified robust changes in general transcript levels and alternative transcript isoforms (Figure 3A). How THO selects its mRNA targets is currently unknown, but its selectivity is remarkably well preserved during metazoan evolution (39–44). Analogous to other model organisms, *thoc-5* or *thoc-7* deficiency does not cause an overt change in total RNA levels (Supplementary Figure S8), but alters the expression of a highly specific subset of genes (<5% of the transcriptome). As expected, we find a significant overlap between *thoc-5* and *thoc-7* mutants in both down- (67%) and upregulated mRNAs (71%) (Figure 3B). None of the shared downregulated transcripts, however, have been implicated in DDR signalling, DNA metabolism or repair (Supplementary Figure S9). Among the shared upregulated transcripts (Supplementary Figure S10) are three factors implicated in DNA metabolism: *atl-1*(∼1.7-fold), *gei-17* (∼1.4-fold) and *rev-1* (∼1.9-fold), possibly reflecting a response to DNA replication stress (45). Previous studies found elevated levels of ATL-1 foci and DNA replication stress-associated ubiquitination in sterile *thoc-2* deletion mutants (33,46). Defective mRNA processing is often associated with R-loop formation, perhaps due the ineffective extraction of nascent RNA away from its DNA template (32). We quantified global R-loop levels using dot blots and found that the genomes of our identified *thoc-2*, *thoc-5* and *thoc-7* mutants indeed have elevated levels of RNA:DNA hybrids (Figure 3C and D). While these R-loops can trigger DNA replication stress and possibly alter DNA repair, the mechanism by which THO deficiency controls NHEJ does not rely on DNA replication (as it occurs in differentiated pharyngeal cells and arrested L1 animals). Detailed analysis of RNA expression changes within individual transcripts confirmed widespread yet highly selective alterations in mRNA transcription, splicing and/or degradation in *thoc-5* or *thoc-7* animals (Supplementary Figure S8). The most frequent RNA processing defects observed among *thoc-5* or *thoc-7* mutants involve skipped exons or retained introns, which supports a role for THO in mRNA maturation (Figure 3E). Multivariate analysis of transcript splicing (MATS) identified 90 skipped exons and 18 retained introns in *thoc-5* mutants, and 27 skipped exons and 10 retained introns in *thoc-7* mutants. More than half of these splicing defects were shared between the two THO mutants, yet none of the shared defects prevents expression of known DNA repair factors (Supplementary Figure S11). Splicing defects often disrupt the reading-frame and thus are potent triggers of PTCs and subsequent NMD activation (47). Transcript analysis at the nucleotide level confirmed robust expression of PTC-bearing mRNAs in the THO mutants (Figure 3F and Supplementary Figure S12). Our unbiased DSB repair screen thus identified viable THO mutants that suffer from a selective DNA repair defect and multiple RNA stresses (including RNA:DNA hybrids and non-sense RNAs).

### PNN-1 and the THO complex are needed for efficient NHEJ

**Figure 4.**
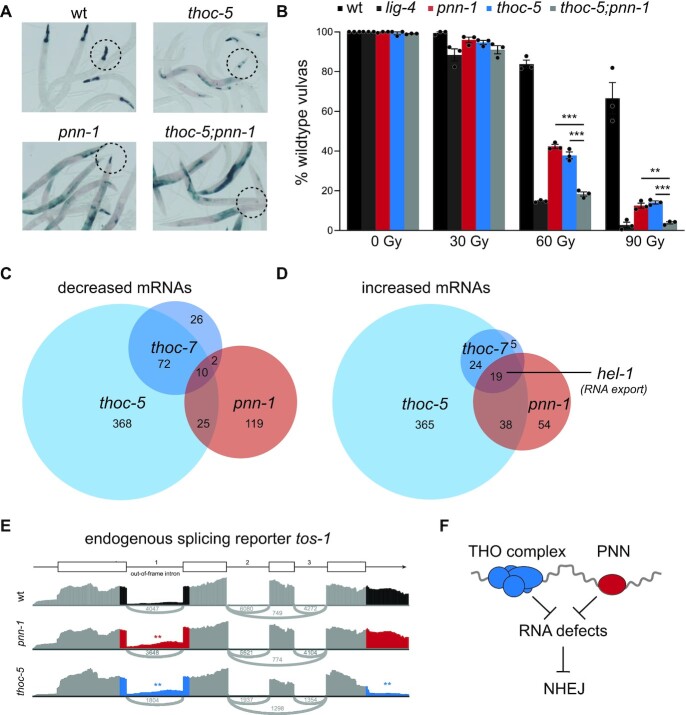
THOC-5 and PNN-1 control RNA processing and NHEJ efficacy. (**A**) Representative pictures of LacZ staining patterns of dual reporter animals. Circles indicates representative pharynges; please note that *thoc-5;pnn-1* double mutants lack LacZ staining of pharynges while showing robust LacZ expression in other somatic tissues, indicative of a selective, highly penetrant NHEJ defect. (**B**) Larvae were challenged with indicated doses of IR and vulva development was scored. Values depict averages of three independent experiments and error bars represent S.E.M. Dots indicate the average of each experiment. Statistical analysis shows significant differences in vulva development (two-tailed *t*-tests *P < 0.05; **P < 0.01, ***P < 0.001). please note that *thoc-5;pnn-1* double mutants show radiosensitivity similar to *lig-4* null mutants. (**C**). Venn diagram of significantly (q ≤ 0.05) downregulated transcripts identified using RNA sequencing. (**D**) Venn diagram of significantly (*q* ≤ 0.05) upregulated transcripts identified using RNA sequencing. One of the nineteen transcripts upregulated in all three mutants, *hel-1*, is highlighted. (**E**) Examples of splicing errors in *thoc-5* and *pnn-1* mutants, enhancing expression of an out-of-frame intron. Gene model of *tos-1* and below simplified sashimi plots for each genotype. Sashimi plots depict relative RNAseq read-depth around the highlighted region of *tos-1*. Asterisks indicate significant alternative splicing events as detected by DEXSeq (*P*_adjusted_ ≤ 0.05). (**F**) Working model; The presence of the THO complex and PNN is necessary to prevent RNA defects that impede NHEJ.

The DSB repair screen yielded one additional mutant (allele *lf159*), which based on epistasis and mapping studies was not affected in known NHEJ genes nor in THO genes. Instead, we found a causal nonsense mutation in *pnn-1* (R186.7), the *C. elegans* ortholog of the mRNA splicing and export factor Pinin/DRS/memA (Supplementary Figure S13). Animals with an independently derived *pnn-1* deletion allele (*ok1872*) confirmed a reduction in NHEJ, a shift towards SSA and hypersensitivity to IR (Figure 4A and B). Comparative analysis using *thoc-5 pnn-1* double mutants showed a mild, yet reproducible additive defect in NHEJ, hinting towards a shared trigger and a common theme between defective RNA processing and NHEJ regulation. To map the degree of functional overlap between PNN-1 and THO components we directly compared the transcriptome profiles of synchronized *pnn-1*, *thoc-5* and *thoc-7* animals by RNAseq. Similar to THO deficiency, *pnn-1* loss affects only a selective subset of the transcriptome (<3%). *pnn-1* mutants share nearly a third of the up-regulated transcripts and half of the down-regulated transcripts with the THO mutants (Figure 4C and D). Interestingly, *thoc-5*, *thoc-7* and *pnn-1* mutants show increased transcript levels of *hel-1*/UAP56 (Figure 4D), which is a well-conserved RNA-DNA helicase known to control mRNA export and interact with THO complex proteins (48). In flies, UAP56 up-regulation is shown to be part of a feedback mechanism that responds to a block in mRNA maturation and export, and human UAP56 is recently shown to resolve RNA–DNA hybrids genome-wide (49,50). Elevated *hel-1*/UAP56 expression thus might indicate a common RNA stress in these animals.

rMATS analysis indeed revealed various splicing defects in *pnn-1* mutants compared to N2 controls, including skipped exons and retained introns (Supplementary Figure S13). Notably, *pnn-1* mutants share more than a third of splicing alterations with *thoc-5* and/or *thoc-7* animals, substantiating a common role for PNN-1 and THO in RNA surveillance (Supplementary Figure S8 and S13). In-depth analysis at the nucleotide level confirmed matching RNA expression changes in *pnn-1* and *thoc-5* mutants; e.g. both mutants show enhanced retention of the first intron of *tos-1*, which is a well-characterized endogenous reporter gene for splicing perturbations (51). These analyses also revealed gene-specific effects, as only THO mutants show an additional defect at the 3’ end of *tos-1* transcripts (Figure 4E). PNN-1 and THO thus have non-identical yet highly similar functions in RNA processing, which parallels their effect on NHEJ. The identification of PNN-1 and THO in NHEJ regulation implies an extensive connection between RNA surveillance and DNA repair and suggests that unprocessed RNA molecules may confound DNA repair (Figure 4F).

### SMG-1 kinase impairs NHEJ in THO deficient animals

**Figure 5.**
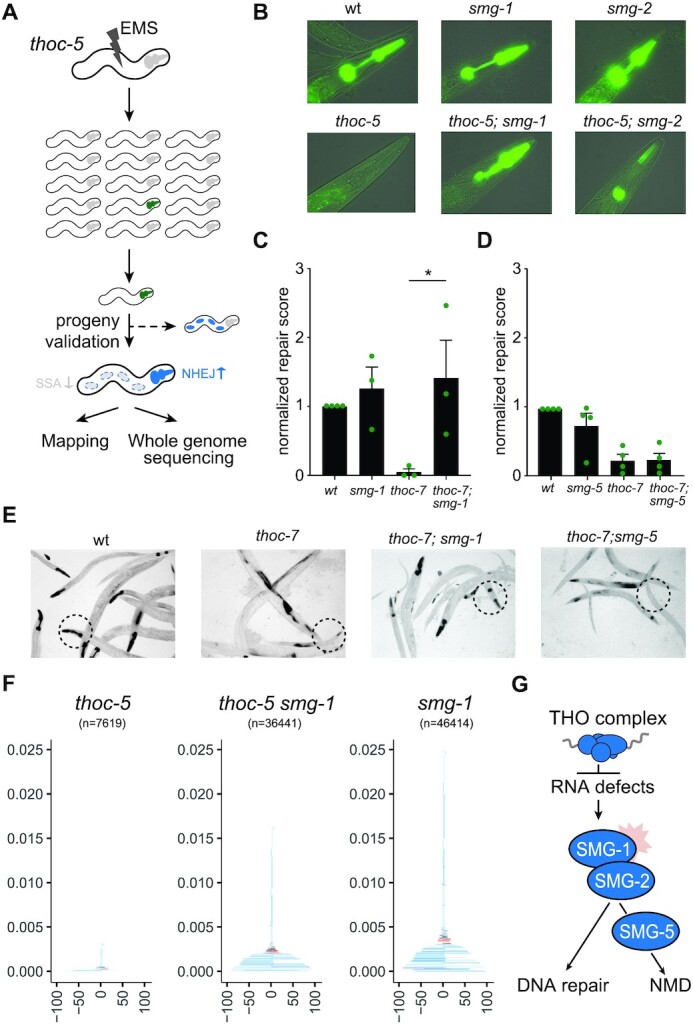
Loss of SMG-1 or SMG-2, but not SMG-5, restores NHEJ in THO deficient animals. (**A**) NHEJ suppressor screen set-up. Genomes of *thoc-5* animals carrying the dual reporter system were mutagenized using ethyl methanesulfonate (EMS). NHEJ activity of the progeny was assessed and GFP-positive worms were isolated. Restored NHEJ activity was verified in *thoc-5* animals using LacZ staining patterns among clonal progeny. NHEJ-proficient candidates were followed up to identify the causal mutation. (**B**) Representative pictures of pharyngeal GFP expression in NHEJ reporter animals. (**C**) Normalized repair score of reporter animals. Average of three populations (*n* > 200) is depicted. Dots indicate the average of each experiment. The repair score is determined by dividing the % of NHEJ-positive animals by the % of SSA-positive animals. Repair scores are normalized to the wildtype control for each independent experiment to correct for LacZ staining and DSB induction efficacy. Error bars represent SEM (two-tailed *t*-tests *P < 0.05; **P < 0.01, ***P < 0.001). (**D**) Normalized repair score of reporter animals. Average of three populations (*n* > 200) is depicted. Dots indicate the average of each experiment. The repair score is determined by dividing the % of NHEJ-positive animals by the % of SSA-positive animals. Repair scores are normalized to the wildtype control for each independent experiment to correct for LacZ staining and DSB induction efficacy. Error bars represent SEM (two-tailed *t*-tests *P < 0.05; **P < 0.01, ***P < 0.001). (**E**) LacZ staining patterns; circles highlight representative pharynges (i.e. NHEJ proficiency). (**F**) Tornado plots depict type and size of mutagenic repair events identified by NGS (n indicates number of mutagenic events). Color code indicates type of event as in Figure 2G. (**G**) Working model; while RNA defects due to THO deficiency activate NMD by SMG-1, SMG-2 and SMG-5, only SMG-1 and SMG-2 have moonlighting functions in DDR and inhibit NHEJ.

To identify the mechanism connecting RNA surveillance to NHEJ we performed an unbiased genetic suppressor screen (Figure 5A). We reasoned that if RNA stress is responsible for an altered DNA damage response, we could potentially restore normal NHEJ levels in THO mutants by mutating potential signalling components.

To this end, we mutagenized *thoc-5* mutant animals that carry the fluorescent reporter system and identified a suppressor mutant that restored NHEJ activity in *thoc-5* deficient animals. The identified suppressor mutation restored I-SceI-dependent GFP fluorescence and alleviated IR sensitivity in *thoc-5* deficient animals (Supplementary Figure S14). Combining positional mapping and whole-genome sequencing revealed a candidate suppressor mutation in *smg-1*, a gene encoding a well-conserved kinase implicated in both NMD and DDR signalling (52) (Figure 5B). The identified *smg-1*(*lf238*) allele causes a nonsense mutation and is predicted to express a severely truncated SMG-1 peptide lacking its well-conserved kinase and FACT domains (Supplementary Figure S1). Notably, SMG-1 loss also fully rescues NHEJ activity in animals bearing a missense mutation in *thoc-*7, indicating that restored NHEJ activity does not rely on non-sense *thoc-5* mRNA, which is a possible NMD substrate (Figure 5C–E). Depleting SMG-1 using RNAi also restored NHEJ activity in *thoc-7* mutant animals and *smg-1* loss did not compromise NHEJ reporter specificity, confirming SMG-1 as a bona-fide NHEJ suppressor (Supplementary Figure S14). Next-generation sequencing of DSB repair footprints revealed restored deletion formation in *thoc-5 smg-1* double mutants compared to *thoc-5* single mutants, including typical repair products of NHEJ (deletions without homology) or TMEJ (deletions with micro-homology and templated inserts) (Figure 5F). Independent phospho-proteome analysis also suggests a major role for SMG-1 in THO deficient animals, as *thoc-5* mutants show increased phosphorylation at >800 unique phosphorylation sites compared to wildtype controls, while their *smg-1* deficient counterparts have <300 unique phosphorylation events (Supplementary Figure S15 and Supplemental Table S1). Although this whole-animal proteomics approach is unable to identify direct SMG-1 targets (i.e. direct phospho-peptides are lost/undetectable in SMG-1 null mutants), it substantiates a key role for SMG-1 in altered signalling in THO mutants. SMG-1 phosphorylates numerous cellular proteins beyond those required for NMD. We thus wondered if the observed crosstalk between RNA surveillance and NHEJ activity requires SMG-1 specifically or NMD activity in general. Genetic dissection of the NMD pathway revealed that SMG-2, the major downstream target of SMG-1 also contributes to NHEJ suppression in THO deficient animals (Figure 5B, Supplementary Figure S16). In contrast, loss of SMG-5, a critical NMD factor that controls the dephosphorylation of SMG-1 targets, did not restore NHEJ activity in THO deficient animals (Figure 5D-E). We also performed RNAi studies and found SMG-1 but not SMG-5 depletion to restore NHEJ in *thoc-7* deficient animals (Supplementary Figure S14). Likewise, NHEJ in *thoc-7* mutants could be fully restored by a deletion allele of SMG-6 but not by a reported null allele of SMG-4 (Supplementary Figure S18). These observations have important implications for the mechanism of NHEJ interference: (i) although NMD substrates are elevated in THO mutants, it is not the stabilisation of a particular NMD substrate that rescues the NHEJ defect, as the latter would also be the case in *smg-4* and *smg-5* animals and (ii) SMG-1 could impair NHEJ via its established NMD targets SMG-2 and SMG-6, which much like SMG-1 have reported functions in both RNA surveillance and DNA integrity (53,54). THO deficiency thus causes a defect in DSB repair, which does not require NMD *per se*, but rather the specific activity of moonlighting NMD factors, such as the PIKK SMG-1 and the DNA/RNA helicase SMG-2 (Figure 5G).

## DISCUSSION

DNA and RNA fulfil very different functional niches within the cell, yet their chemical structures are highly similar. Due to their analogous structure, many proteins bind both DNA and RNA, which predicts a high level of crosstalk between DNA and RNA regulation. Indeed, DNA damage changes the activity of many RNA-binding factors and thus also dictates how a cell processes RNA (55). It is, however, unclear whether the reverse is also true. Can defects in RNA processing feed into the DDR and change the way a cell repairs DNA? The latter could have major consequence, as it would translate stochastic RNA expression changes into permanent scars in the genome. Here, we identified two well-conserved proteins, SMG-1 and SMG-2, that in an animal system cause a short-circuit between RNA processing and DNA repair.

**Figure 6.**
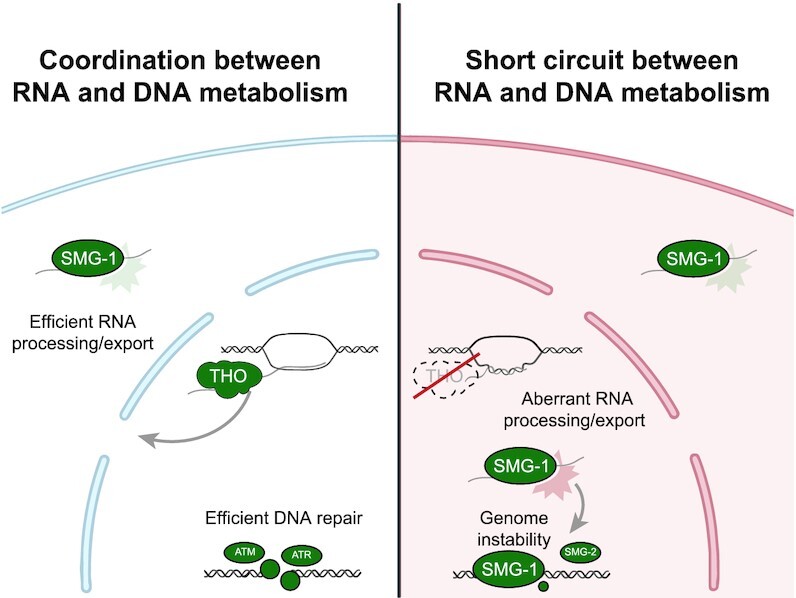
Crosstalk between RNA defects and DNA repair. In wildtype situations, the THO complex processes newly transcribed RNA, enabling efficient export and preventing RNA:DNA hybrids. SMG-1 functions as a NMD mediator to remove defective transcripts. In situations where efficient RNA processing is impaired, like in THO mutant cells, we hypothesize that defective transcripts accumulate and hyperactivate SMG-1. Deregulation of this moonlighting kinase alters the DNA damage response, which leads to a decrease in NHEJ efficiency. Thus, in conditions that cause aberrant RNA processing, protein moonlighting can create short-circuits between RNA and DNA maintenance. This figure was created using Biorender under an academic license (https://biorender.com).

We created and used animal models to monitor DSB repair in somatic tissues and identified highly conserved RNA binding proteins that cause defective NHEJ (i.e. Pinin and the THO complex). Both THO and Pinin associate with spliceosomes and have been implicated in pre-mRNA processing and mRNA export (56,57). Intriguingly, defective THO complex function is known to result in genome instability in various species, including yeast, worms and humans, which in part could be explained by its role in preventing the formation of RNA:DNA hybrids that hinder DNA replication throughout the genome (33,34). Our previous study on *met-2 set-25* double mutant animals, however, indicates that accumulation of high levels of R-loops does not compromise NHEJ *per* se (58), and our current findings in terminally differentiated muscle cells demonstrate that either loss of PNN-1 or THO function can alter DSB repair in the absence of DNA replication. Indeed, the new *thoc-7*, *thoc-5* and *thoc-2* alleles identified here are compatible with fertility, while *thoc-2* null mutants are sterile due to severe replication defects (33), further strengthening the notion that the role of THO in NHEJ regulation can be separated from its established role in R-loop prevention and DNA replication fidelity. We find that PNN-1 and THO deficiency cause a strikingly similar change in RNA metabolism genome-wide, including altered transcript levels and splicing defects. Importantly, animals lacking PNN-1 or THO also shared multiple features of compromised NHEJ activity, including reduced NHEJ products, elevated SSA and IR hypersensitivity. Subsequent genetic screening revealed the signalling factor linking RNA processing to DNA repair: the moonlighting kinase SMG-1. SMG-1 was first identified to control RNA surveillance via NMD but later was found to also respond to DNA damage and control cell cycle arrest and p53 expression (52,59,60). These latter functions in DNA surveillance are shared by the related kinases ATM and ATR, suggesting a possible ancestral role of PIKKs in genome stability. Interestingly, both ATM and ATR have recently been shown to be activated not only by broken DNA but also by R-loops (35,36), suggesting that intertwined DNA-RNA signalling might be a common theme among PIKKs. We also would like to emphasize that the key players identified here in *C. elegans* (THOC-5, THOC-7 and SMG-1) are also found in humans, but are not present in budding yeast, arguing that the crosstalk between NMD and NHEJ might be a trait of multicellular organisms. Our results indicate that pathological conditions causing RNA stress can derail DSB repair via hyperactivation of the PIKK SMG-1. Probable triggers for SMG-1 activity in THO mutants include elevated levels of non-sense RNAs and R-loops. Based on the notion that PTC-containing RNAs are potent activators of SMG-1 activity, and the observation of increased PTC-containing RNAs (but not DSBs) in the here-studied THO mutant animals, we consider it likely that PTC-containing RNAs act upstream of SMG-1 in modulating NHEJ efficacy. However, *smg-5*, *smg-4* and *smg-1* mutants are reported to accumulate PTC-containing RNAs, yet only the latter efficiently restores NHEJ in THO mutants, which supports the notion that PTC-containing RNAs alone are not sufficient to perturb NHEJ. Similarly, these data imply that the DNA repair defect is not caused by NMD activity *per se* and thus does not require degradation of a particular non-sense RNA. We believe that our data is best explained by a signalling route in which THO loss cause aberrant mRNA, which via SMG-1 results in a NHEJ defect. We also find that SMG-2, a well-established phosphorylation target of SMG-1, impairs NHEJ in THO mutants. Interestingly, the human ortholog of SMG-2, UPF1, is a multifunctional DNA/RNA helicase with roles in NMD and DNA replication (53). Recent *in vitro* studies revealed that SMG-2/UPF1 binds both DNA and RNA, and can act as a highly progressive helicase on both substrates (61,62). Our results suggest that the dual roles of SMG-1 and SMG-2 in RNA and DNA surveillance can distort DNA repair and trigger genome instability, particularly in conditions that impair the processing and nuclear export of RNA (Figure 6).

How SMG-1 suppresses NHEJ activity at the molecular level and if such crosstalk affects human development are key questions for future research; recent progress on Cryo-EM structures of SMG-1 complexes as well as the development of selective SMG-1 kinase inhibitors will provide new clues and therapeutic opportunities to this end (63). Because our genetic screen for NHEJ factors was not saturated (e.g. we did not identify *lig-4*mutants), future screening efforts may also lead to the identification of additional regulators of SMG-1 activity and/or DSB repair; while we observed a SMG-1-dependent increase in phosphorylation in THO mutants, our method did not allow us to study the direct targets of SMG-1. Identifying the phosphorylation status of known DNA repair enzymes in THO deficient contexts and assessing the dependency of their phosphorylation sites on SMG-1 may provide mechanistic detail on how SMG-1 affects DNA repair.

In this study, we have used reported null alleles of NMD genes to investigate their impact on NHEJ efficiency but have not quantitatively compared NMD activity in different genetic backgrounds to NHEJ functionality, which may provide insight into the relative contribution of individual NMD factors to NHEJ regulation. Novel tools that enable detection and/or regulation of R-loop levels in vivo, such as inducible and/or tissue-specific RNAse H expression systems, could also help assessing causal roles of R loops in different DNA repair deficiencies.

Here, we provide proof-of-concept that crosstalk exists between RNA surveillance and DNA repair and propose that stochastic defects in RNA processing can permanently shape the DNA landscape via deregulation of SMG-1. Notably, genetic conditions causing elevated SMG-2/UPF1 phosphorylation have recently been linked to severe developmental retardation, microcephaly and variable facial and organ malformations, emphasizing the health risks associated with deregulated SMG-1 activity (64). Given that many RNA processing proteins have multiple functions, also in processes not related to NMD or DNA repair (e.g. PNN-1 controls splicing and cell adhesion), we predict that signalling crosstalk via moonlighting proteins is abundant *in vivo* and might have a major impact on basic physiology and pathology. Recent estimates indeed suggest that 78% of moonlighting proteins are involved in human diseases, which is significantly higher than the 18% disease association for proteins in general (65). We predict that the recent advances in unbiased genetic screening in both animal models and human cells will contribute to the discovery of hidden signalling connections and reveal additional vulnerabilities caused by moonlighting proteins.

## DATA AVAILABILITY

Raw sequences have been made publicly available at NCBI SRA (accession code PRJNA678136 and PRJNA674486). Sequencing data for N2 wild-type, *brc-1* and *lig-4* animals were published previously and can be found at NCBI SRA (accession codes PRJNA260487 and PRJNA599297. The phosphoproteomics data have been deposited to the ProteomeXchange Consortium via the PRIDE partner repository with the dataset identifier PXD022616.

## Supplementary Material

gkac472_Supplemental_Files
