# Supplementary material for: THO complex deficiency impairs DNA double-strand break repair via the RNA surveillance kinase SMG-1
Source: Nucleic Acids Res. 2022 Jun 7;50(11):6235–50. doi: 10.1093/nar/gkac472 (PMC9226523; doi:10.1093/nar/gkac472)
Supplement: gkac472_Supplemental_Files [file gkac472_supplemental_files.zip › 20220407_Kamp_Lemmens_2022_Supplemental.pdf]

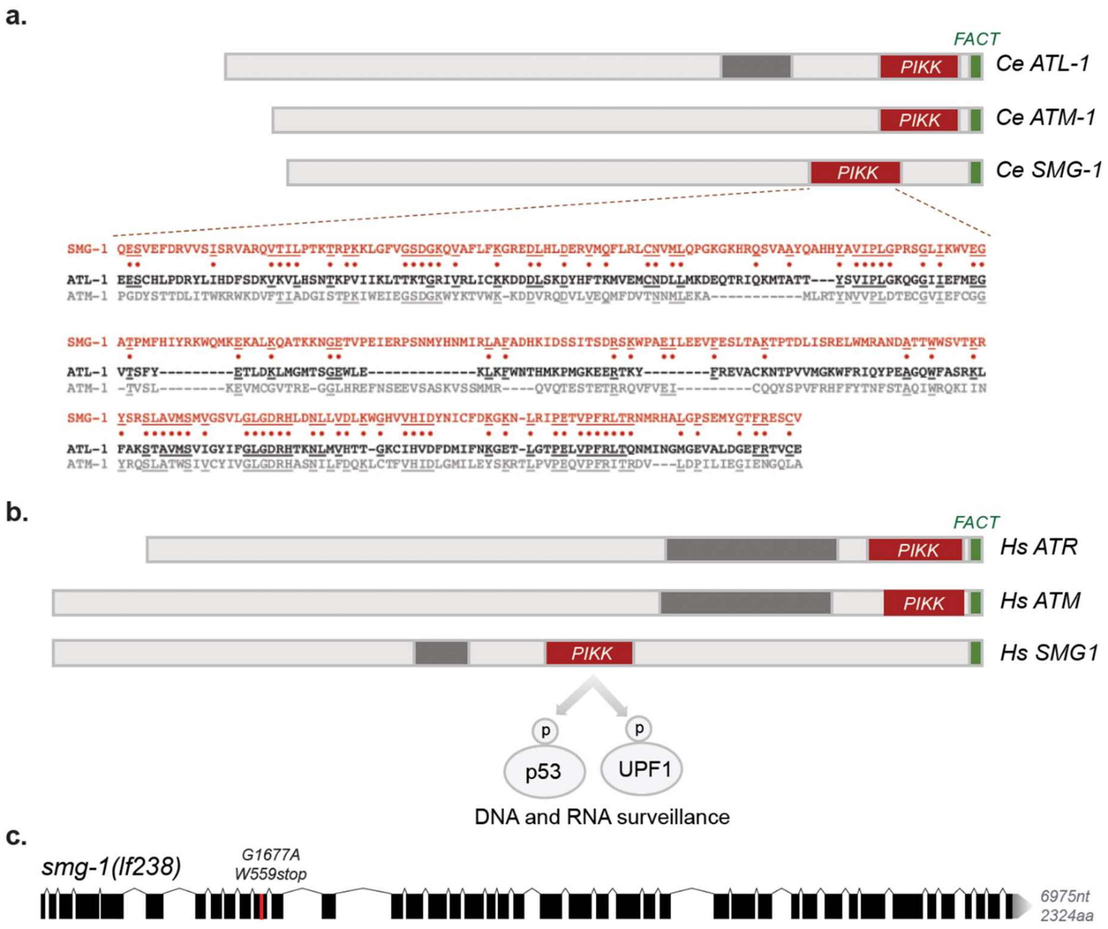

Figure S1. SMG-1 shares conserved domains and phosphor-targets with DDR kinases

a. Schematic diagram of protein domain structures of *C. elegans* PIKK-family kinases ATL-1, ATM-1 and SMG-1. Relative position of FACT domains (green), PIKK catalytic domains (red) and FAT domains (dark grey) are indicated. Lower panel shows sequence alignment of PIKK domains of the indicated proteins. Dots highlight homologous residues between SMG-1 (red) and ATL-1 (black) or ATM-1 (grey). **b.** Schematic diagram of protein domain structures of human PIKK-family kinases ATR, ATM and SMG1. Relative position of FACT domains (green), PIKK catalytic domains (red) and FAT domains (dark grey) are indicated. Arrows indicate two established SMG1 phosphorylation targets involved in the DNA damage response (p53) or NMD (UPF1). **c.** Gene model and newly identified non-sense allele of *smg-1*.

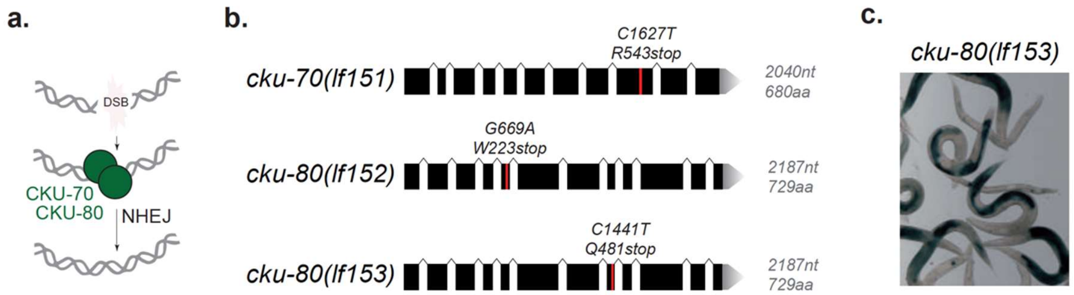

Figure S2. Identified *cku-70* and *cku-80* alleles verify in vivo NHEJ screen

a. Schematic diagram of conserved role of CKU-80/CKU-70 heterodimer in DSB recognition and repair via NHEJ. **b.** Gene models and newly identified alleles of *cku-70* and *cku-80*. **c.** LacZ expression patterns of *cku-80* deficient dual reporter animals. Synchronized animals were heat-shocked for 120 minutes at L1 stage to introduce I-SceI-induced DSBs and NHEJ/SSA reporter expression is detected in adults.

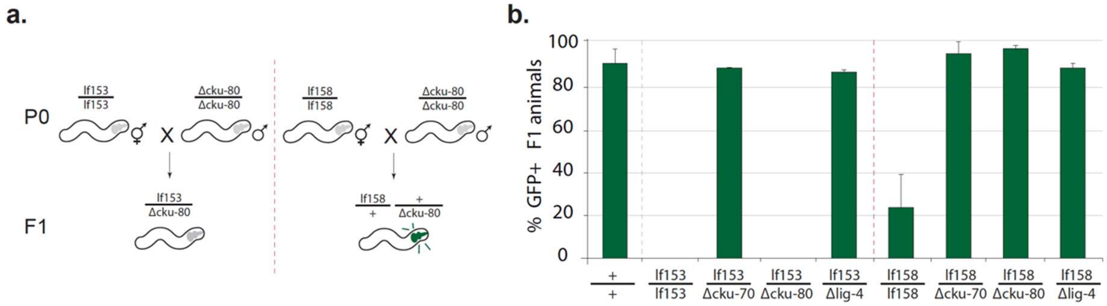

Figure S3. Example complementation analysis revealing non-canonical NHEJ mutants

a. Example of crossing scheme used for complementation analysis. Established and uncharacterized NHEJ mutants are crossed and trans-heterozygous F1 progeny is analysed for somatic NHEJ activity. **b.** Quantification of GFP-positive pharynges in trans-heterozygous F1 cross progeny, heat-shocked for 120 minutes to introduce I-SceI-induced DSBs and measured in adults. Average percentage of GFP-positive pharynges of three independent F1 populations is depicted.

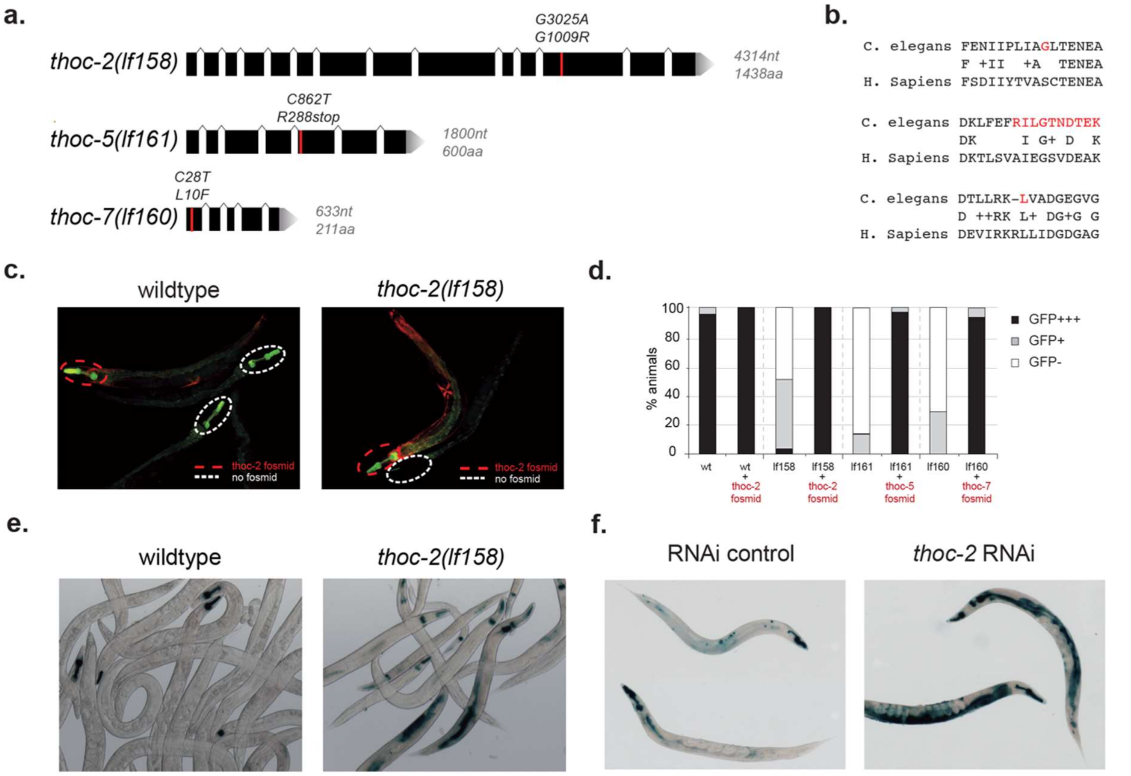

Figure S4. Identified *thoc-2*, *thoc-5* and *thoc-7* alleles and causal effect validation

a. Gene models and newly identified alleles of *thoc-2*, *thoc-5* and *thoc-7*. Right panel depicts amino acid context and conservation of THO mutations. **b.** Amino acid context and conservation of identified THO mutations (affected amino acids in red). **c.** Complementation analysis using fosmid arrays carrying wild-type THO genes and mCherry expression markers. Representative picture of synchronized populations of wildtype and lf158 animals with (red circle) or without (white circle) *thoc-2* fosmid array; animals were heat-shocked for 180 minutes at L1 stage. **d.** Histogram shows quantification of GFP-positive pharynges in adults of the different genetic backgrounds. NHEJ activity was restored to wild-type levels by complementing the THO mutants with functional THO genes. Average percentage of GFP-positive pharynges of three independent populations (n>150) is depicted. **e.** LacZ expression patterns of *thoc-2* deficient dual reporter animals and wildtype parental controls. Animals were heat-shocked for 120 minutes at L1 stage to introduce I-SceI-induced DSBs and NHEJ/SSA reporter expression is detected in adults. **f.** Representative pictures of LacZ staining patterns in synchronized populations of dual reporter animals fed bacteria carrying empty vectors of thoc-2 RNAi vectors. L4 animals were heat-shocked for 120 minutes to introduce I-SceI-induced DSBs and reporter expression is detected the next day. Depletion of *thoc-2* caused a three-fold increase in LacZ positive bodies (SSA).

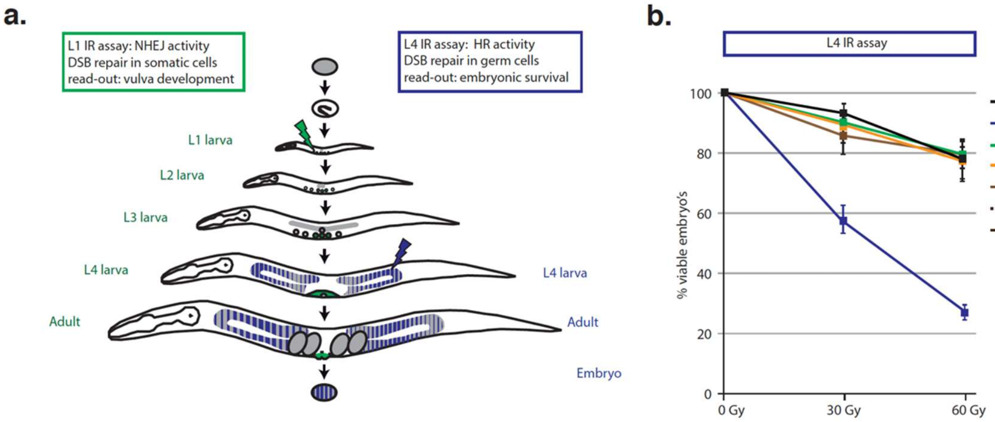

Figure S5. Germ cells deficient for *thoc-5* or *thoc-7* are not hypersensitive to IR **a.** Schematic representation of two different IR assays in the context of the C. elegans life cycle and challenged tissues. While the L1 assay measures IR-resistance of arrested vulva precursor cells and mainly reflects NHEJ activity, the L4 assay measures IR-resistance of germ cells and typically reflects HR activity. **b.** L4 assay; L4 animals were challenged by the indicted dose of IR and percentage of viable progeny is plotted. Values depict the average of three independent experiments and error bars represent S.E.M.

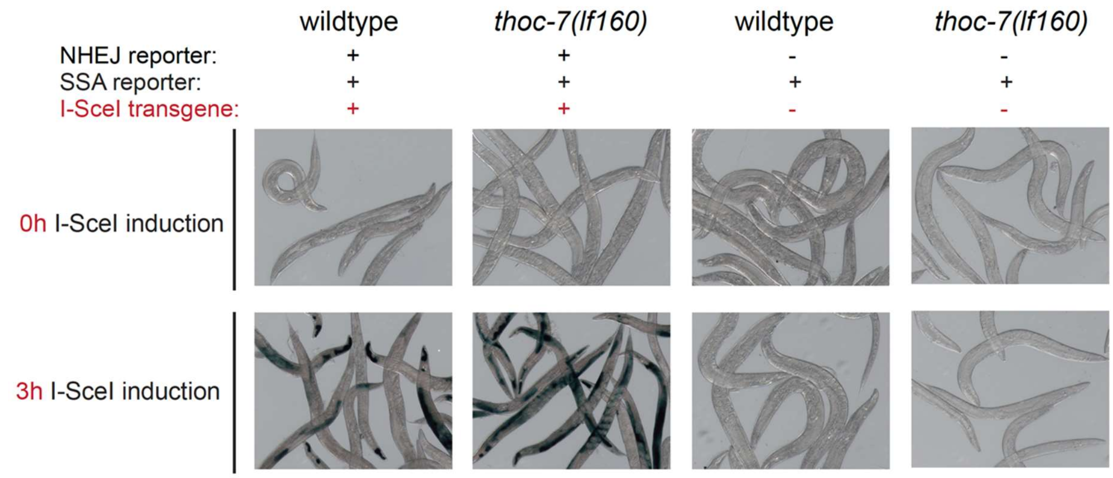

Figure S6. Increased SSA in *thoc-7* mutants requires I-SceI induced DSB formation

LacZ staining patterns in synchronized populations of animals carrying either the dual SSA/NHEJ reporter system or the SSA reporter only, the latter lacking the heat-shock inducible I-SceI expression transgene. Synchronized animals were heat-shocked 0 or 180 minutes at L1 stage and LacZ expression was detected in adults. Increased somatic LacZ (SSA) in *thoc-7* mutants required the presence of the I-SceI transgene and heat-shock-driven I-SceI expression.

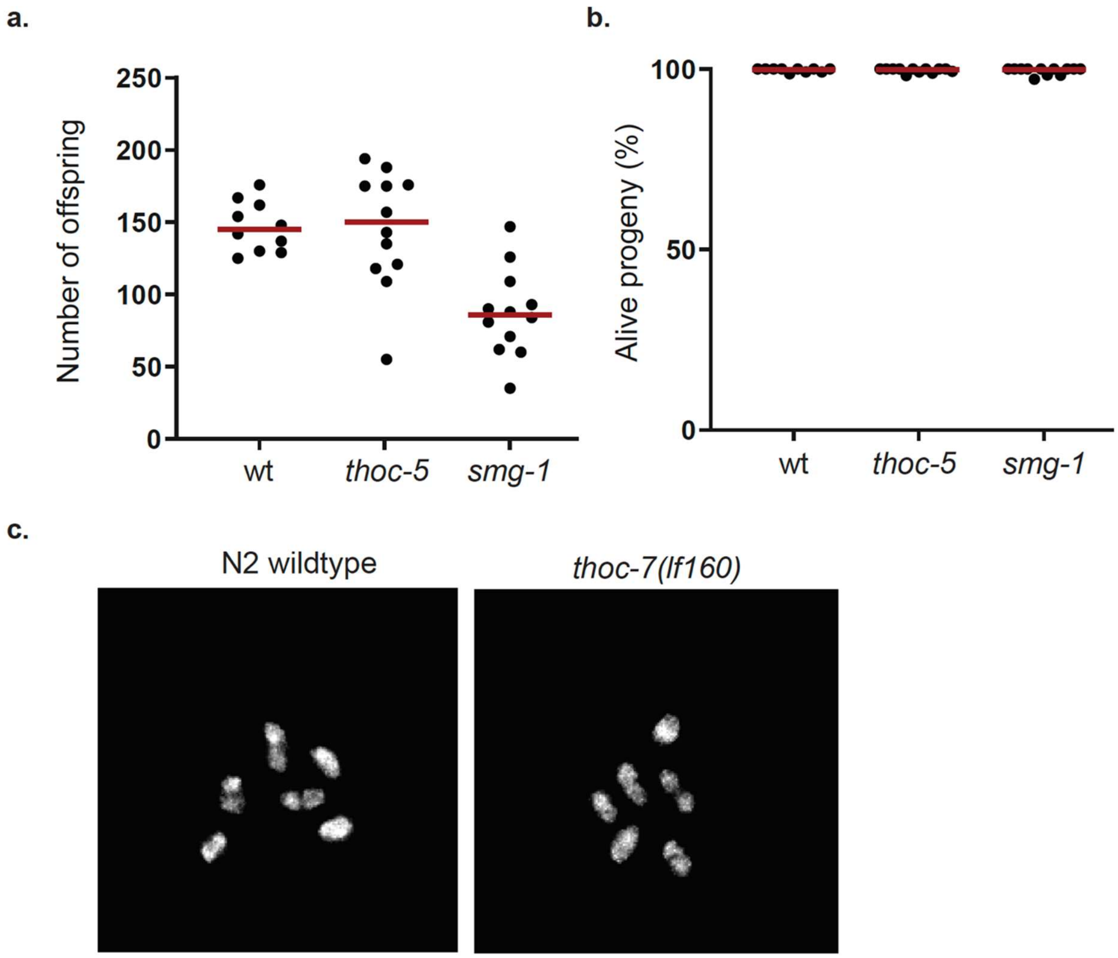

Figure S7. Fecundity and meiotic recombination in *thoc-5* or *thoc-7* mutant animals

a. Brood size quantification. Each dot depicts the number of offspring generated by a single worm. Lines depict median. **b.** Embryonic survival quantification. Each dot depicts the percentage of alive offspring of the brood generated by a single parent. **c.** DAPI-stained diakinesis nuclei (germline staining protocol as in [7]). The presence of six intact bivalents is indicative of proficient meiotic recombination.

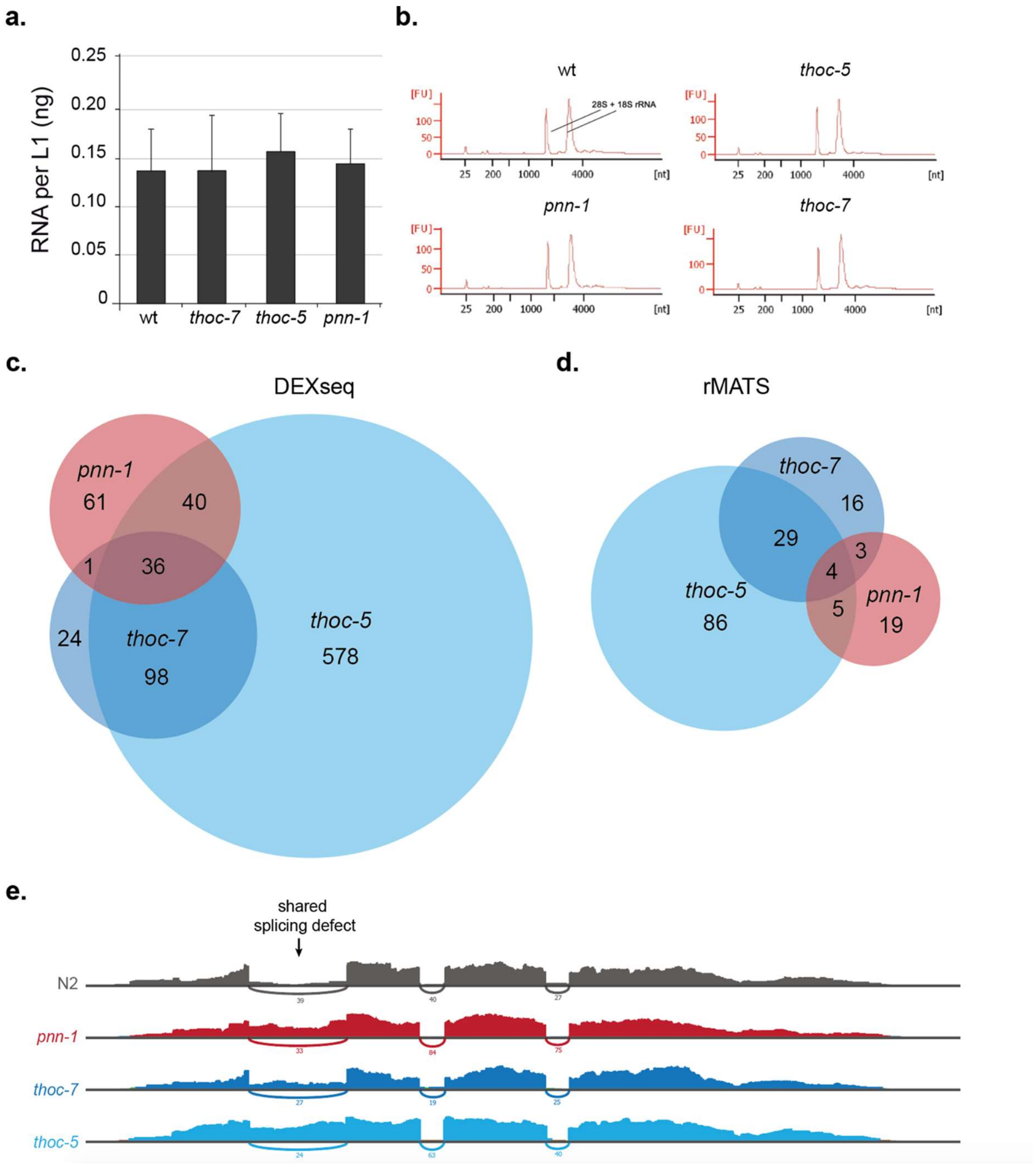

Figure S8. RNAseq reveals shared splicing defects in THO and PNN-1 mutants
a. Average total RNA yield per L1 animal determined from four independent L1 populations of the indicated genotype. **b.** RNA quality and size distribution determined using Agilent Eukaryote Total RNA Nano Lab-on-a-Chip. Panels depict representative RNA profiles for each genotype. Two well-defined peaks of 28S and 18S rRNA are indicative of intact RNA. **c.** Venn diagram of exon expression changes compared to N2 controls identified by DEXseq **d.** Venn diagram of alternative splicing events compared to N2 controls identified by rMATS. **e.** Example of shared intron retention in *nhr-263* mRNA identified by iREAD. Simplified sashimi plots depict relative RNAseq read-depth for each genotype. The number of junction reads in *nhr-263* are indicated at the arcs connecting the pair of exons.

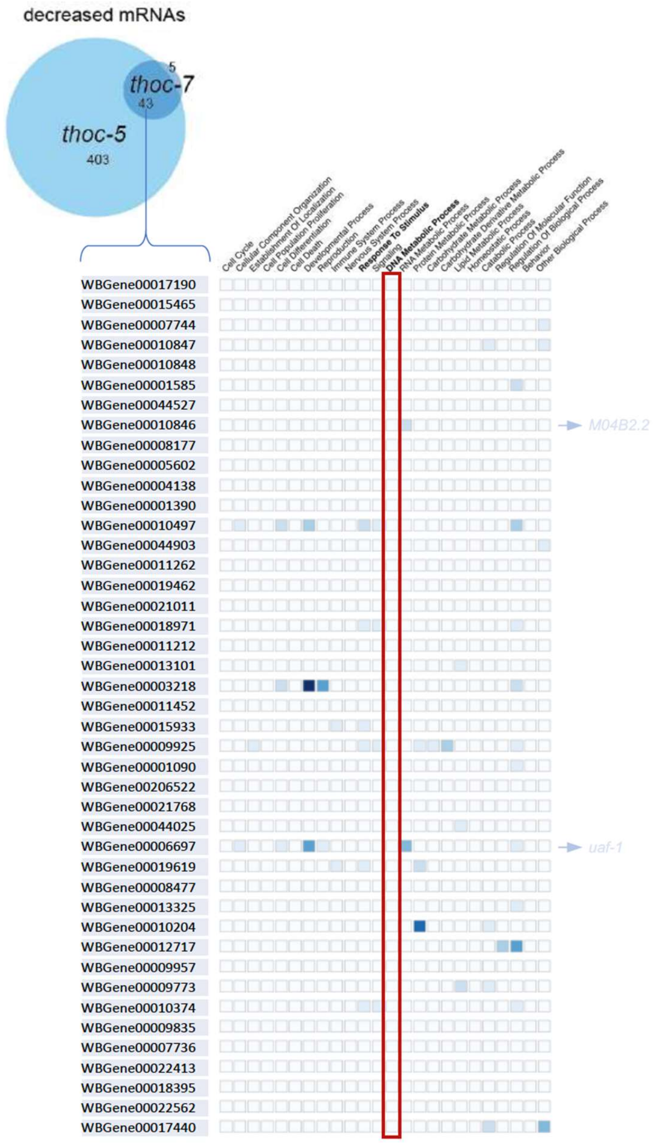

Figure S9. Gene Ontology ribbons of down-regulated genes in THO mutants
Table visualizing the GO terms/biological processes annotated to the 43 genes of which mRNA expression was significantly decreased in *thoc-5* and *thoc-7* mutants compared to wildtype controls, detected by DEseq. Darker blue boxes indicate terms with the most annotations; white boxes represent terms that are not annotated for this gene. Red box highlights annotation to GO term ‘DNA metabolic process’, which includes DNA repair and DNA replication. On the right, genes annotated to the biological processes ‘Response to Stimulus’ or ‘DNA metabolic process’ are highlighted with light blue or red arrows, respectively.

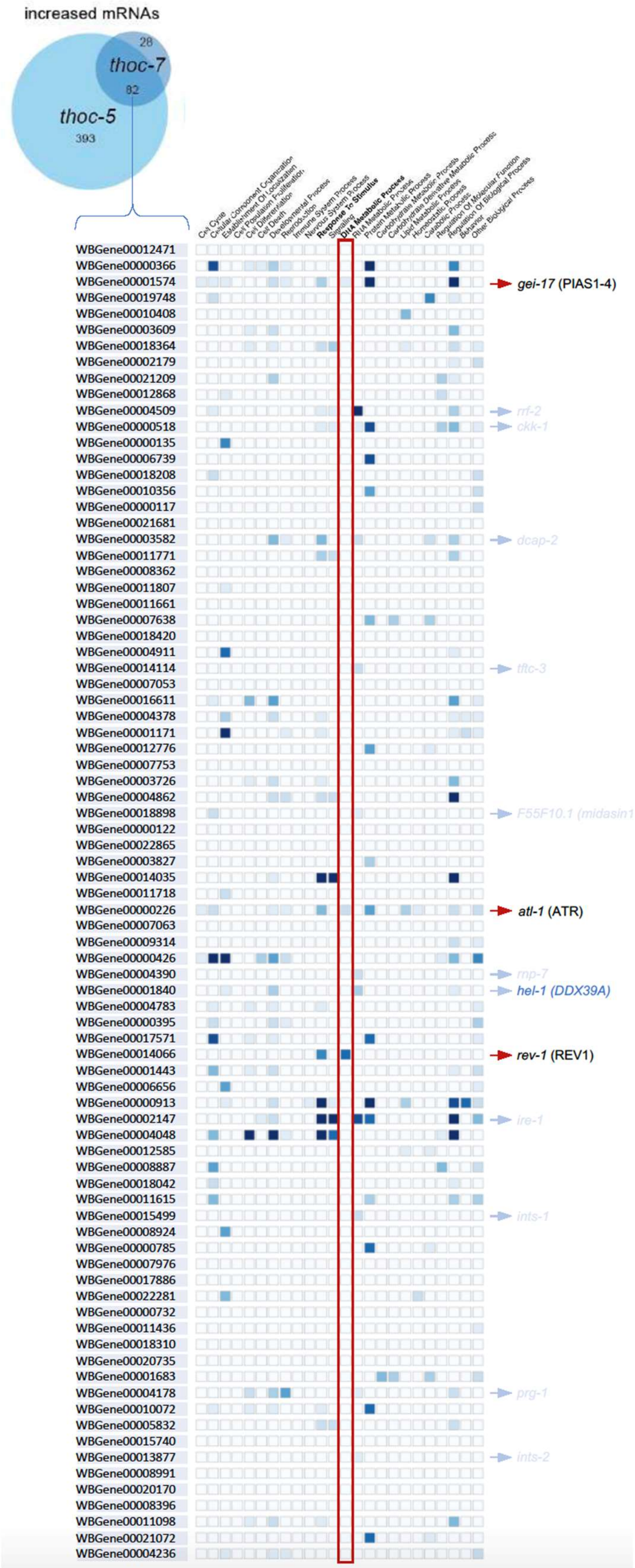

Figure S10. Gene Ontology ribbons of up-regulated genes in THO mutants

Table visualizing the GO terms/biological processes annotated to the 82 genes of which mRNA expression was significantly increased in *thoc-5* and *thoc-7* mutants compared to wildtype controls, detected by DEseq. Labels/annotation as in Figure S9.

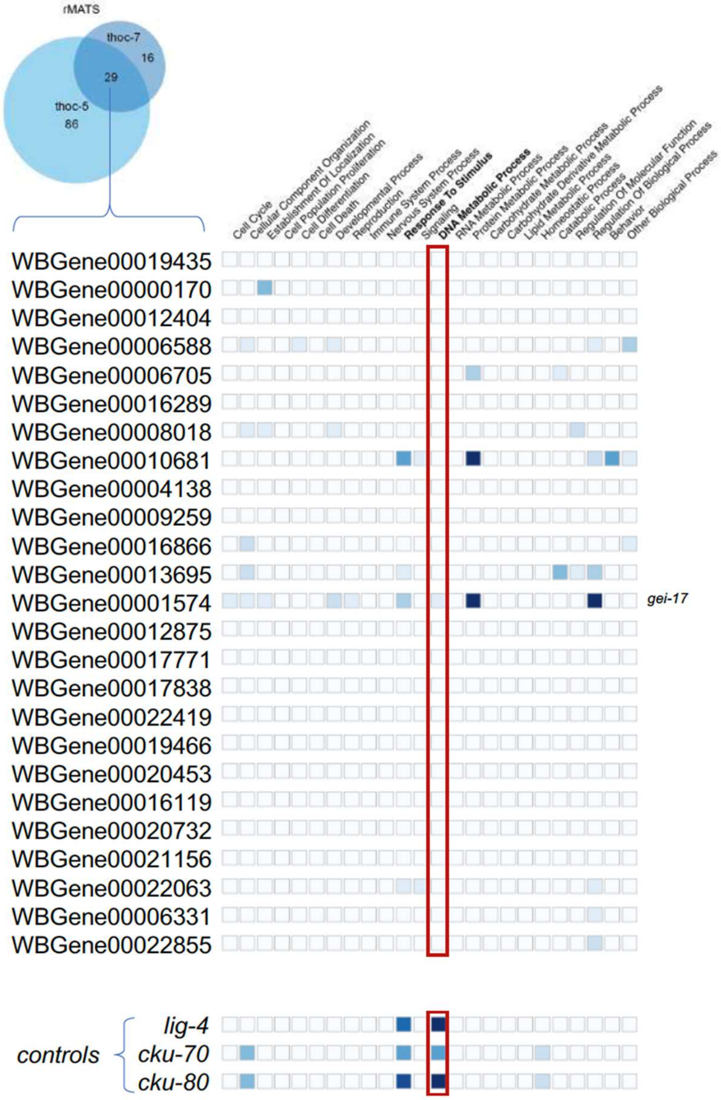

Figure S11. Gene Ontology ribbons of mis-spliced transcripts in THO mutants
Table visualizing the GO terms/biological processes annotated to the 29 genes of which mRNA expression was significantly increased in *thoc-5* and *thoc-7* mutants compared to wildtype controls, detected by rMATS. Darker blue boxes indicate terms with the most annotations; white boxes represent terms that are not annotated for this gene. Red box highlights annotation to GO term ‘DNA metabolic process’, which includes DNA repair and DNA replication. Control genes with known roles in NHEJ are depicted below. Although *gei-17* is implicated in DNA repair and DNA replication stress responses, only a minor fraction of *gei-17* transcripts is alternatively spliced and its overall expression is increased 1.4 fold.

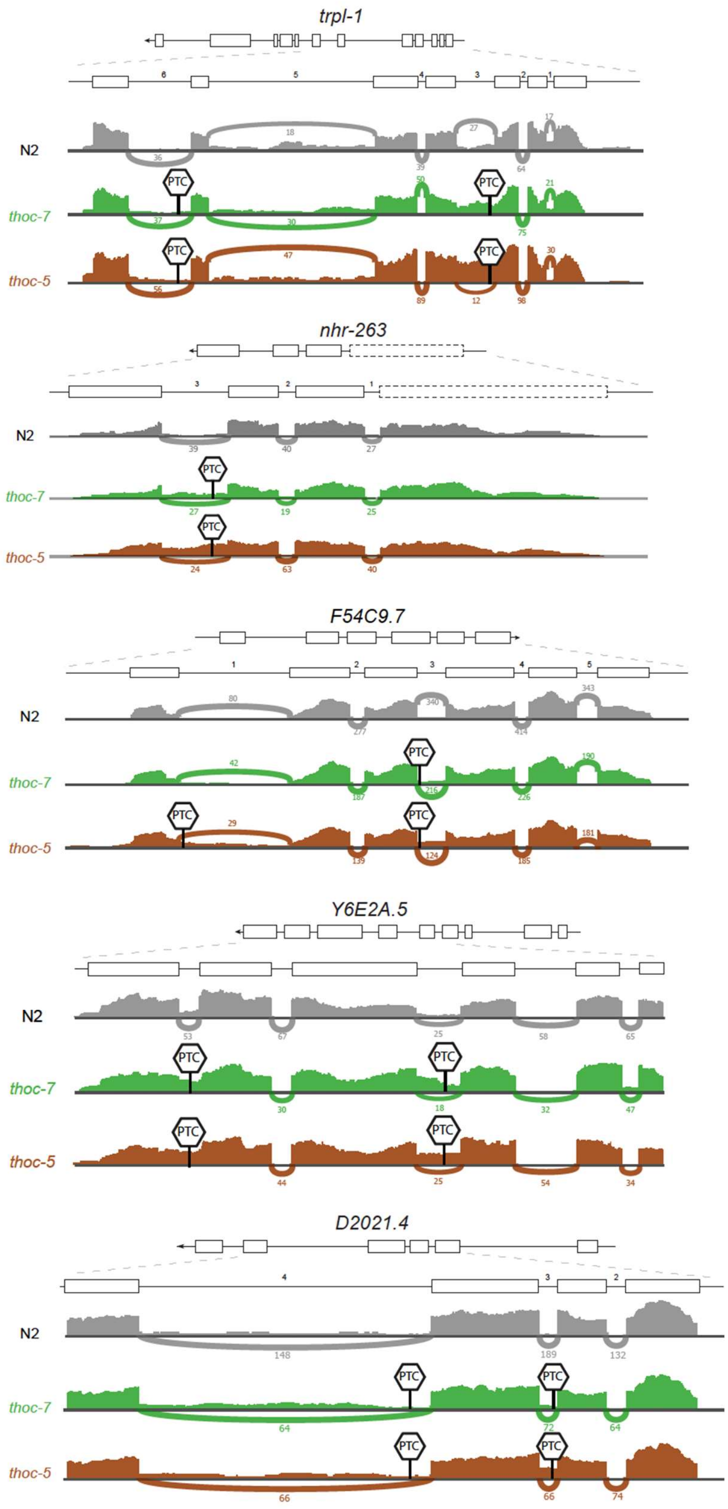

Figure S12. Examples of PTC-bearing transcripts in *thoc-5* and *thoc-7* mutants
Five genes exemplifying increased non-sense transcripts in *thoc-5* and *thoc-7* mutants. For each gene, simplified gene models and sashimi plots are depicted. Sashimi plots indicate relative RNAseq read-depth for each genotype and junction reads are shown as arcs connecting the pair of exons. PTC sign indicates position of a predicted premature stop codon.

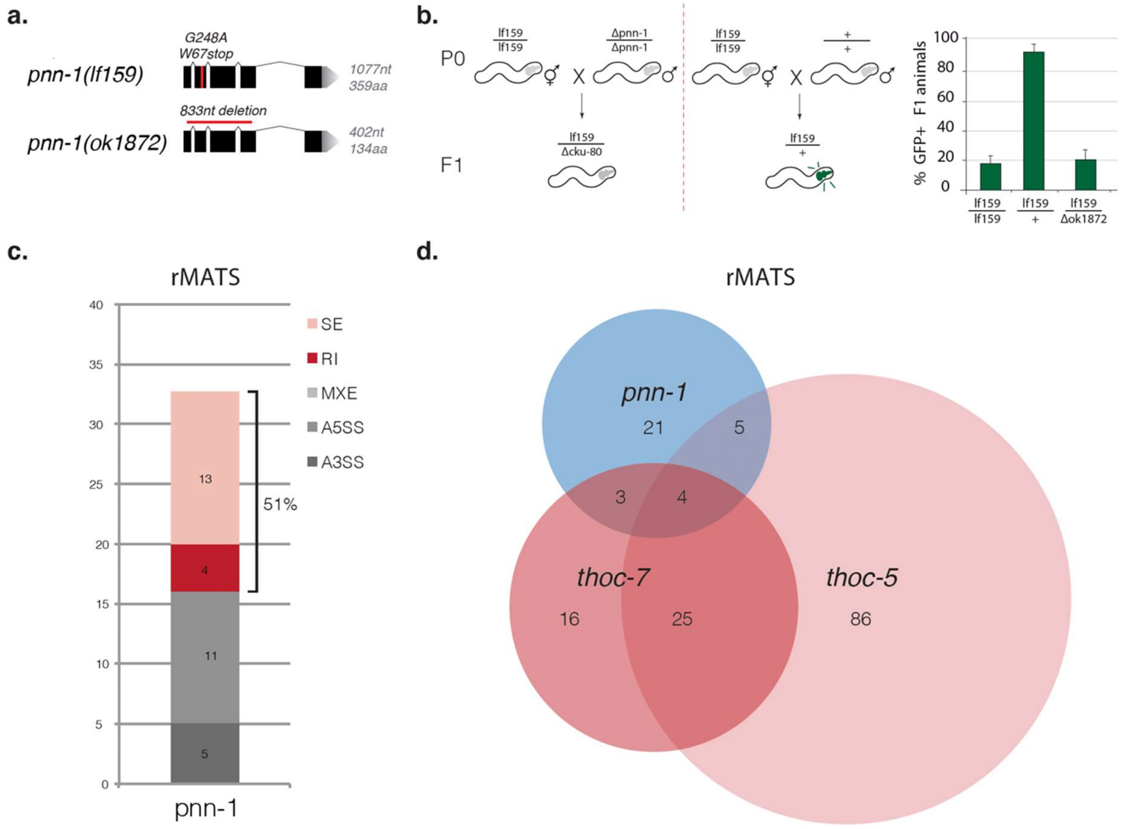

Figure S13. Validation of NHEJ and RNA splicing defects in *pnn-1* deficient animals

a. Gene models and loss-of-function alleles of *pnn-1*. **b.** Crossing scheme used for complementation analysis. Newly identified NHEJ mutants (homozygous for *lf159* allele) are crossed with either wildtype animals or *pnn-1* deletion mutants. trans-heterozygous F1 progeny is analysed for somatic NHEJ activity. Right graph shows quantification of GFP-positive pharynges in F1 cross progeny, heat-shocked for 120 minutes and measured in adults. **c.** Bar chart indicates number of splicing events detected and altered in *pnn-1* deletion mutants compared to wildtype controls. Splicing events as identified by rMTAS: SE, skipped exon; RI, retained intron; MXE, mutually exclusive exon; A5SS, alternate 5′ splice site; A3SS, alternate 3′ splice site. **d.** Venn diagram depicts overlap among differentially spliced genes in *pnn-1*, *thoc-7* and *thoc-5* mutants. Alternatively splicing of C53C7.5, *pdxk-1*, W02F12.4 and F07B10.4 is shared by all three mutants.

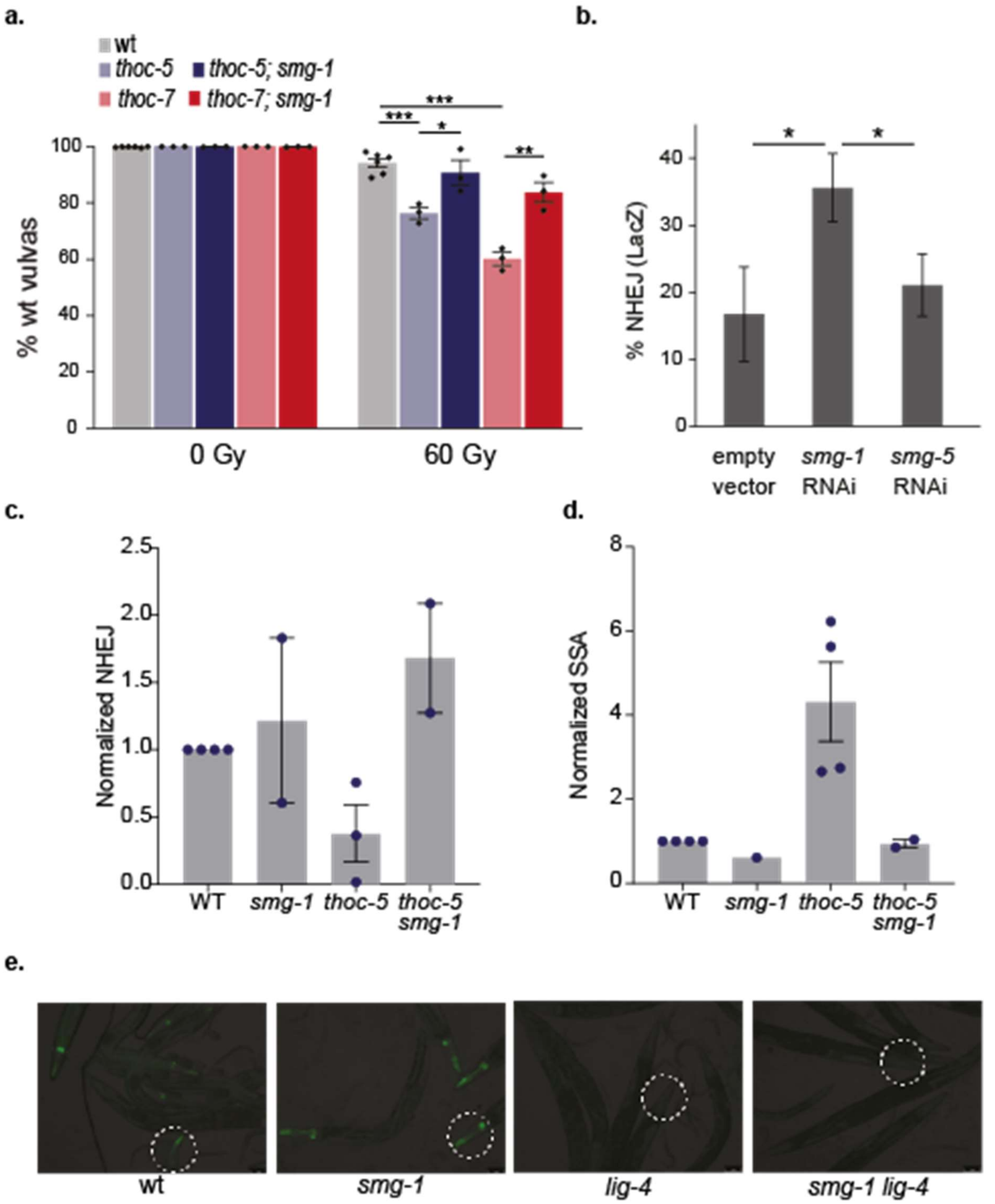

Figure S14. Confirmation of SMG-1 as a suppressor of classical NHEJ **a.** The newly identified smg-1 allele (lf238) alleviates IR hypersensitivity of THO mutants. L1 animals were challenged with indicated doses of IR and vulva development was scored. Values depict averages of independent experiments and error bars represent S.E.M. Dots indicate the average of each experiment (two-tailed t-tests *P<0.05; **P<0.01; ***P<0.001) **b.** NHEJ activity in *thoc-7* mutants after RNAi-mediated knockdown of *smg-1* or *smg-5*. Bars indicate average fraction of LacZ-positive pharynges of three populations. Error bars depict standard deviation (two tailed t-tests, *P<0.05). **c.** Quantification of LacZ-positive pharynges. Dual reporter animals are heat-shocked for 120 minutes at L1 stage to induce I-SceI expression and

LacZ reporter expression is analysed in adults. Dots indicate the average of independent populations. Error bars represent SEM. **d.** Quantification of LacZ-positive somas. Dual reporter animals are heat-shocked for 120 minutes at L1 stage to induce I-SceI expression and LacZ reporter expression is analysed in adults. Dots indicate the average of independent populations. Error bars represent SEM. **e.** GFP expression in heat-shocked dual reporter animals for each indicated genotype. Representative pharynges are encircled. Loss of *smg-1* does not restore GFP expression in *lig-4* mutants, indicating that *smg-1* deficiency does not alter the specificity of the NHEJ reporter.

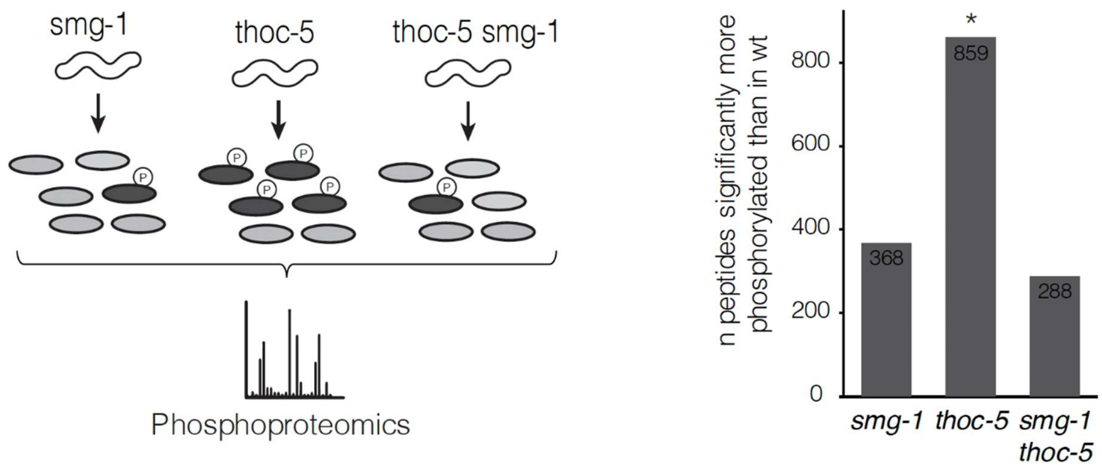

Figure S15. Altered protein phosphorylation upon thoc-5 and/or smg-1 loss Schematic of proteomics approach (left). Proteins lysates were obtained from asynchronous populations of *smg-1*, *thoc-5* and *thoc-5; smg-1* mutants, along with wildtype controls, and enriched for phosphopeptides. For each genotype, unique phosphorylation events were detected and quantified using mass spectrometry. Bar graph represents the quantification of the amount of peptides significantly more phosphorylated than in wild-type worms (permutation test, *P<0.05).

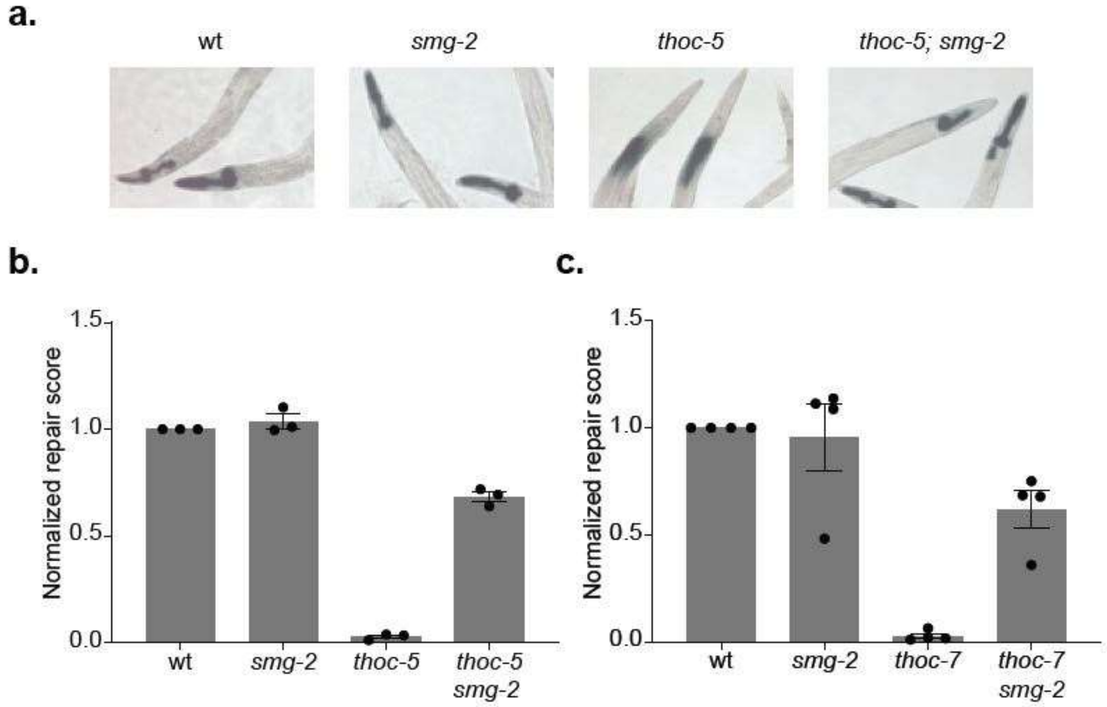

Figure S16. SMG-2 deficiency restores NHEJ activity in *thoc-5* and *thoc-7* mutant animals

a. LacZ expression patterns of heat-shocked dual reporter animals (quantified in b.). **b.** Quantification of LacZ-positive pharynges. Dual reporter animals are heat-shocked for 120 minutes at L1 stage to induce I-SceI expression and LacZ reporter expression is analysed in adults. Dots indicate the average of independent populations. Error bars represent standard deviation. **c.** Quantification of LacZ-positive pharynges. Dual reporter animals are heat-shocked for 120 minutes at L1 stage to induce I-SceI expression and LacZ reporter expression is analysed in adults. Dots indicate the average of independent populations. Error bars represent SEM.

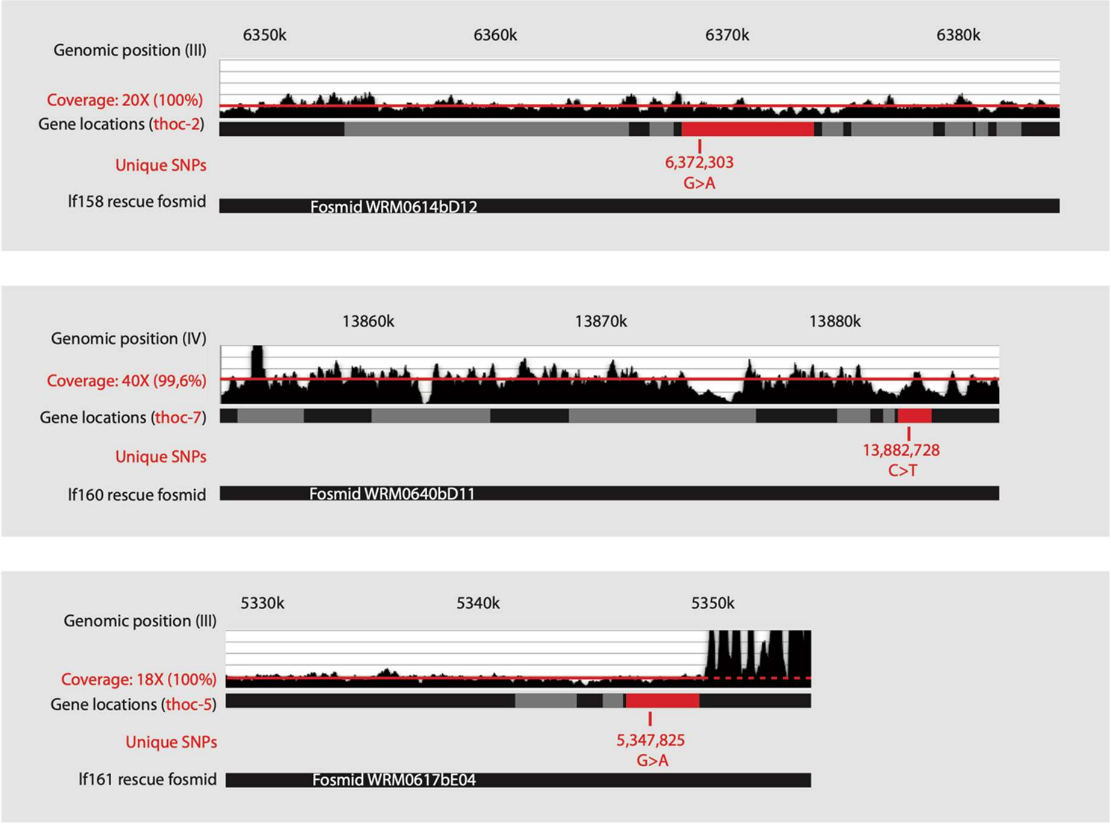

Figure S17. Coverage and genomic context of regions spanned by rescue fosmids described in figure S4

Unique non-synonymous SNPs in the genomic regions spanned by the injected fosmids (highlighted in red). Despite high sequence coverage, no other unique variants than those in the THO genes were found in the respective NHEJ mutants, advocating that we specifically complemented THO deficiency despite the use of 2-4kb fosmids.

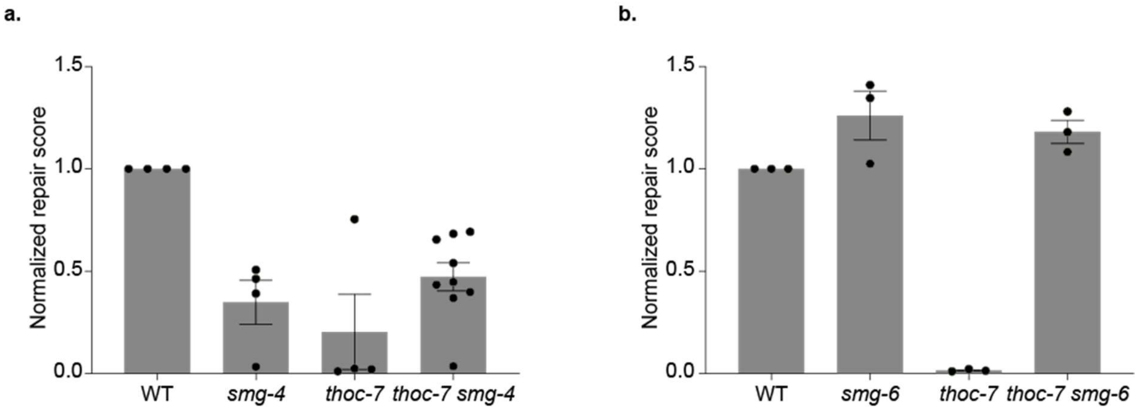

Figure S18. SMG-6 deficiency, but not SMG-4 deficiency, restores NHEJ activity in *thoc-7* mutant animals

a. Normalized repair score of reporter animals. Dual reporter animals are heat-shocked for 120 minutes at L1 stage to induce I-SceI expression and LacZ reporter expression is analysed in adults. Dots indicate the average of independent populations. Error bars represent SEM. **b.** Normalized repair score of reporter animals. Dual reporter animals are heat-shocked for 120 minutes at L1 stage to induce I-SceI expression and LacZ reporter expression is analysed in adults. Dots indicate the average of independent populations. Error bars represent SEM.
